# Human pathways in animal models: possibilities and limitations

**DOI:** 10.1093/nar/gkab012

**Published:** 2021-02-01

**Authors:** Nadezhda T Doncheva, Oana Palasca, Reza Yarani, Thomas Litman, Christian Anthon, Martien A M Groenen, Peter F Stadler, Flemming Pociot, Lars J Jensen, Jan Gorodkin

**Affiliations:** Center for non-coding RNA in Technology and Health, University of Copenhagen, 1871 Frederiksberg, Denmark; Department of Veterinary and Animal Sciences, University of Copenhagen, 1870 Frederiksberg, Denmark; Novo Nordisk Foundation Center for Protein Research, University of Copenhagen, 2200 Copenhagen, Denmark; Center for non-coding RNA in Technology and Health, University of Copenhagen, 1871 Frederiksberg, Denmark; Department of Veterinary and Animal Sciences, University of Copenhagen, 1870 Frederiksberg, Denmark; Novo Nordisk Foundation Center for Protein Research, University of Copenhagen, 2200 Copenhagen, Denmark; Translational Type 1 Diabetes Research, Steno Diabetes Center Copenhagen, 2820 Gentofte, Denmark; Department of Immunology and Microbiology, University of Copenhagen, 2200 Copenhagen, Denmark; Exploratory Biology, LEO Pharma A/S, 2750 Ballerup, Denmark; Center for non-coding RNA in Technology and Health, University of Copenhagen, 1871 Frederiksberg, Denmark; Department of Veterinary and Animal Sciences, University of Copenhagen, 1870 Frederiksberg, Denmark; Animal Breeding and Genomics, Wageningen University & Research, 6700 Wageningen, The Netherlands; Center for non-coding RNA in Technology and Health, University of Copenhagen, 1871 Frederiksberg, Denmark; Bioinformatics Group, Department of Computer Science; Interdisciplinary Center for Bioinformatics; German Centre for Integrative Biodiversity Research (iDiv) Halle-Jena-Leipzig; Competence Center for Scalable Data Services and Solutions Dresden-Leipzig; Leipzig Research Center for Civilization Diseases; and Centre for Biotechnology and Biomedicine, University of Leipzig, 04107 Leipzig, Germany Max Planck Institute for Mathematics in the Sciences, 04103 Leipzig, Germany; Institute for Theoretical Chemistry, University of Vienna, 1090 Vienna, Austria; Facultad de Ciencias, Universidad Nacional de Colombia, Bogotá D.C., Colombia The Santa Fe Institute, 87501 Santa Fe, NM, USA; Center for non-coding RNA in Technology and Health, University of Copenhagen, 1871 Frederiksberg, Denmark; Translational Type 1 Diabetes Research, Steno Diabetes Center Copenhagen, 2820 Gentofte, Denmark; Faculty of Health and Medical Sciences, University of Copenhagen, 2200 Copenhagen, Denmark; Center for non-coding RNA in Technology and Health, University of Copenhagen, 1871 Frederiksberg, Denmark; Novo Nordisk Foundation Center for Protein Research, University of Copenhagen, 2200 Copenhagen, Denmark; Center for non-coding RNA in Technology and Health, University of Copenhagen, 1871 Frederiksberg, Denmark; Department of Veterinary and Animal Sciences, University of Copenhagen, 1870 Frederiksberg, Denmark

## Abstract

Animal models are crucial for advancing our knowledge about the molecular pathways involved in human diseases. However, it remains unclear to what extent tissue expression of pathways in healthy individuals is conserved between species. In addition, organism-specific information on pathways in animal models is often lacking. Within these limitations, we explore the possibilities that arise from publicly available data for the animal models mouse, rat, and pig. We approximate the animal pathways activity by integrating the human counterparts of curated pathways with tissue expression data from the models. Specifically, we compare whether the animal orthologs of the human genes are expressed in the same tissue. This is complicated by the lower coverage and worse quality of data in rat and pig as compared to mouse. Despite that, from 203 human KEGG pathways and the seven tissues with best experimental coverage, we identify 95 distinct pathways, for which the tissue expression in one animal model agrees better with human than the others. Our systematic pathway-tissue comparison between human and three animal modes points to specific similarities with human and to distinct differences among the animal models, thereby suggesting the most suitable organism for modeling a human pathway or tissue.

## Graphical Abstract

**Graphical Abstract:**
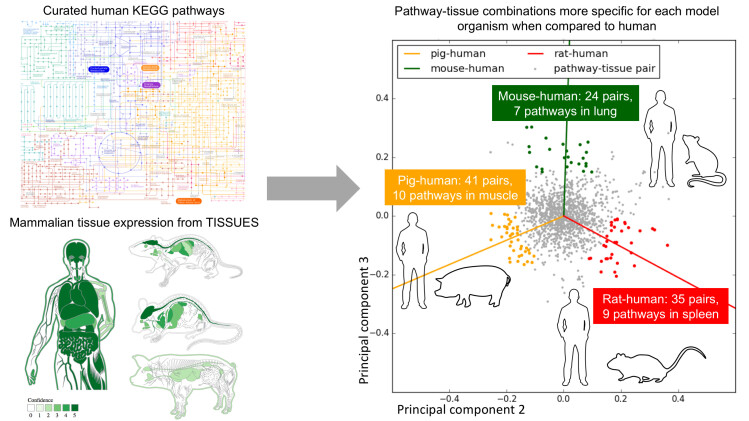
Pathway-tissue comparison between human and three animal models.

## INTRODUCTION

Animal models play important roles in understanding human diseases. A main concern in using animal models for studying human diseases is the fundamental, but not clearly proven, assumption that the genes, pathways and diseases in model organisms are comparable to those of human. No systematic studies have tested this assumption at the tissue expression or pathway levels, although specific tissues or pathways have been compared (1,2). Even with closely related species, such as human and chimpanzee, it might not be trivial to identify the subtle differences in pathway regulation, which may be critical for disease modeling or drug design (3,4). Moreover, the most used animal models, often a rodent such as mouse or rat or an ungulate as the pig, are much more distantly related to human.

Model animals are extensively used to dissect underlying mechanisms of human diseases and to develop new treatments (5), but this is not trivial to do as recently demonstrated (6–9). For example, the most important class of drug metabolizing enzymes, the cytochrome P450 protein family, differs greatly between rodents and humans, both in terms of substrate specificity and multiplicity of the different cytochrome P450 subfamilies (10). For this reason, mice and rats are poor model organisms for testing the effects of drugs that undergo first-pass metabolism in the liver. By contrast, the cytochrome P450 protein family in pig represents a more promising model of human drug metabolism (11). Studies of diseases in animal models cannot be performed without first establishing the physiological pathway regulation in a specific tissue of the healthy animal. It is also important to know to what extent the pathway regulation is the same as in healthy humans, since complex diseases are usually associated with alterations in the activity of one or more pathways (12,13).

Even though many genes between human and model animals are highly similar in both sequence and function, their regulation and interplay can differ. While many databases and resources characterize genes in numerous species (14–16), most pathway annotation efforts focus on human, and most of those available for animal models are thus derived from human pathways (17,18). In the case of primary protein–protein interaction databases that contain experimentally determined physical interactions (19–21), very little data is available for animal models. On the other hand, integrative databases such as STRING (22,23) and IID (24) can provide more comprehensive annotations of the interplay between genes in animal models. These databases combine data from multiple resources, spanning interactions from the primary databases, text mining of biomedical literature, and orthology transfer from other organisms. However, because orthology transfer is used to construct such databases, it is not meaningful to subsequently compare human and animal pathways in order to identify similarities and differences between them. For that, organism-specific data on the pathways is needed, which is missing in current databases. Whereas organism-specific pathway annotations and interactions are scarce, expression data is available for many relevant model organisms.

We present the first systematic comparison of human and animal pathway activity for three specific model organisms (mouse, rat and pig), and aim to facilitate researchers in prioritizing animal models for human disease modeling. Key limitations in establishing disease-specific animal models include incomplete pathway annotation in animals and lack of knowledge of organism-specific pathway regulation. However, we show that it is possible to derive the animal pathways by orthology-based transfer of their human counterparts and study their regulation using organism-specific data such as gene expression. We thus map tissue expression data from healthy individuals onto the established derived animal pathways. Tissue expression data has already been compiled for human, mouse, rat, and pig through the TISSUES database (25). However, comparing human and the animal models is still challenging because of differences in the amount and quality of data available for the different organisms. Although we cannot identify animal pathways that deviate from the human version due to lack of organism-specific pathway data, we can detect similarities and differences in pathway activity between human and the animal models. We highlight several pathways, which, for a given healthy tissue, show better agreement in expression between human and one animal model but not the others.

## MATERIALS AND METHODS

### Genomes and gene annotations

Gene numbers for the genomes of human, mouse, rat and pig were extracted from the Ensembl release 95 websites (26) for each organism in January 2019 (Table 1). These correspond to genomes GRCh38 (human), GRCm38 (mouse), Rnor_6.0 (rat) and Sscrofa11.1 (pig). GENCODE numbers (27) were reported in Supplementary Table S1 for human GENCODE 30 (08.04.19) and mouse GENCODE M21 (08.04.19).

**Table 1.** Data availability per resource and organism (human, mouse, rat and pig)

Resource/annotation	Human	Mouse	Rat	Pig
Ensembl genome annotations				
Coding genes	20 418	22 619	22 250	22 452
Non-coding genes	22 107	15 795	8 934	3 250
eggNOG mammalian orthology				
Coding genes assigned to orthologous groups	86.7%	84.3%	76.4%	82.2%
Mentions in biomedical literature				
Organism	–	1 824 080	1 629 280	133 937
Gene	–	1 304 170	734 243	57 230
Gene Ontology annotations				
Experimental	107 301	89 360	49 281	817
Author statement	48 894	4 760	3 396	27
Inferred	86 785	170 033	188 718	47 225
Electronic	74 049	44 022	40 559	101 074
High-scoring STRING protein-protein interactions				
Experimental	18 069	1 304	920	1 266
Experimental transferred	12 713	22 030	39 381	32 312
TISSUES expression data				
Experimental datasets	4	4	3	3
Tissues covered by experimental data	20	20	12	20

The number of coding and non-coding genes for the assemblies of human, mouse, rat, and pig in Ensembl release 95 are reported. From the eggNOG v4.5 orthology database, we report the percentage of genes from each organism that are assigned to a mammalian orthologous group. Text mining of all PubMed abstracts and a subset of full text articles available from PMC provided the number of publications that mention each organism and its genes. We grouped the most recent Gene Ontology annotations into four categories based on their evidence codes and counted the number of annotations for each group in each organism. High-scoring protein–protein interactions from the STRING v10.5 database (overall confidence score above 0.7) were counted. For the TISSUES 2.0 database of mammalian expression, the number of experimental datasets supporting the 21 main tissues is reported together with the number of tissues covered by these datasets. See Supplementary Table S1 as well as Methods for more details.

### Orthology relationships

To identify the orthology relationships between the genes of the studied organisms, we made use of the public resource eggNOG v4.5 (evolutionary genealogy of genes: Non-supervised Orthologous Groups) (28). It provides >190 000 orthologous groups (OGs) of proteins for 2031 organisms at different taxonomic levels and is based on Ensembl release 70. We used the 26 253 OGs at the mammalian level (mOGs). When comparing two or more organisms, it is important to keep in mind that there are different sets of OGs that can be used. Thus, depending on the analysis, one or the other option can be more suitable: (a) OGs that have *at least one* protein for *any* organism, (b) OGs that contain *at least one* protein for *each* organism, (c) OGs with *exactly one* protein for *any* organism, (d) OGs with *exactly one* protein for *each* organism.

At the mammalian eggNOG level, there are 8665 mOGs, which contain exactly one protein for each of the four considered organisms (human, mouse, rat and pig), and 11 500 mOGs, which contain more than one protein for each of the four organisms. When we only require pair-wise relationships, the number of mOGs is larger (see Table 2). For the comparison of annotations and the pathway transferability, we used the mOGs with at least one protein for each organism in the compared pairs. For the pathway–tissue analysis, we focused on the set of 11 500 mOGs that contain at least one protein for each of the organisms.

**Table 2.** Pair-wise overlap of annotations for human–mouse, human–rat, human–pig and mouse–rat

Resource/annotation	Human–mouse	Human–rat	Human–pig	Mouse–rat
eggNOG mammalian orthology				
Common 1-to-1 groups	12 736	11 038	10 916	12 157
Common groups	15 094	13 429	13 573	14 155
Gene Ontology annotations				
Experimental	15 215	4 680	133	3 754
Author statement	1 433	494	2	193
Inferred	50 473	39 740	18 751	64 879
Electronic	25 632	12 430	15 054	13 937
High-scoring STRING protein-protein interactions				
Experimental	537	72	672	66
Experimental transferred	10 422	6 705	9 290	10 859
TISSUES expression data				
Tissues covered by experimental data	19	12	20	12

For each of the selected resources, appropriate features are highlighted. For the eggNOG orthology resources, the number of common (1-to-1) mammalian orthologous groups are reported. For the Gene Ontology annotations and the high-scoring (overall confidence score above 0.7) STRING protein–protein interactions, the number of associations shared between a pair of organisms was determined using the eggNOG mammalian orthology. For the TISSUES 2.0 database, the number of tissues (out of the 21 main tissues) covered by an experimental dataset in both organisms are reported.

### Mentions in biomedical literature

Our in-house text mining software tool called *tagger* runs every week on the whole corpus of more than 31 million PubMed abstracts and the Open Access subset of full-text articles available from PMC (29). In order to determine how often each organism and its genes are mentioned in the biomedical literature, we downloaded the corresponding files on 25 May 2020 from http://download.jensenlab.org/ (30). For each organism, we reported the number of unique PubMed entries (abstracts or full-text articles) in which the organism name occurs based on the file *organism_textmining_mentions.tsv*. To count the number of publications, in which genes of a specific organism occur, we downloaded the separate file for mouse, rat and pig (e.g. *mouse_textmining_mentions.tsv*), which contains a list of PubMed identifiers for each gene of this organism. In these files, a publication is assigned to a gene in an organism if both the gene and the organism were mentioned in the same publication according to *tagger*. In Table 1, we report the number of unique PubMed identifiers assigned to each gene. Since researchers often do not explicitly write in a publication that they study human specifically, and this would thus have to be inferred from the context, the numbers for publications mentioning human or human genes would be inaccurate. For this reason, we refrained from including these in Table 1.

### Gene Ontology annotations

To investigate the coverage of functional annotations, all Gene Ontology (GO) annotations were retrieved on 25 May 2020 from the GO FTP server (31). Each GO annotation has a code assigned to it, which describes how it was determined. The codes were divided into four different groups: (i) *Experimental*: supported directly by an experiment, including high-throughput methodologies (EXP, HAD, HEP, HMP, IDA, IEP, IPI, IGI, IMP); (ii) *Author statement*: based on statements by the authors in the cited reference (TAS, NAS); (iii) *Inferred*: derived from phylogenetic (IKR, IBA) and computational (ISS, RCA, ISO, ISA, ISM) analysis as well as inferred by curators (IC); (iv) *Electronic*: automatically generated and not reviewed electronic evidence (IEA). For each organism and group, we counted the number of unique pairs of GO terms assigned to a protein (see Table 1). The overlap of GO term annotations between pairs of organisms was determined by mapping the annotated genes to their corresponding mOGs and determining the intersection of pairs of GO term and mOGs between the organisms (see Table 2).

### STRING protein–protein interactions

Protein–protein associations were retrieved from STRING, a database of known and predicted protein–protein interactions (22). Raw STRING data (divided into orthology-transferred and original interactions) was downloaded from STRING v10.5. Each file contains the interacting genes (ENSEMBL IDs) and the confidence for each evidence (between 0 and 1), whereas evidences are divided into the original data and the *transferred* interactions (by orthology). To compare the available, high-confidence, not predicted interactions, we considered the *experimental* and *experimental transferred* interactions for each organism. For each interaction type, we counted the number of interactions that have a confidence score ≥0.7 (Table 1) or ≥0.4 (Supplementary Table S1). The overlap of interactions between each pair of organisms was determined by mapping each interacting gene to the corresponding mOG. An interaction was considered overlapping between two organisms if the pairs of interacting genes were in the same mOGs.

### Tissue expression data

For this analysis, we used data from the TISSUES database, which contains gene–tissue associations for human, mouse, rat and pig (25). The database integrates multiple sources of evidence, including transcriptomics data covering all four species, proteomics data only for human, manually curated annotations from UniProt and associations mined from the scientific literature. Importantly, the expression data has been processed such that it is comparable across all sources of evidence and across organisms through a scoring scheme. For each gene–tissue association in each organism, there is an integrated confidence score based on all evidence types. For consistency, the tissue evidence is further summarized into tissue labels, which are based on Brenda Tissue Ontology terms (32).

We retrieved all gene–tissue associations with their corresponding experimental and integrated confidence scores on 26 January 2018. From the 21 tissues, we focused on the seven tissues that are covered by at least two transcriptomics datasets: heart, kidney, liver, nervous system, muscle, lung and spleen. Even though the TISSUES database provides unified confidence scores, the amount and quality of available tissue data varies a lot between organisms due to, for example, study bias. This influences the range of confidence scores as can be seen in the distributions of confidence scores for each tissue (Supplementary Figures S1 and S2). In order to define whether a gene is expressed or not, we calculated organism- and tissue-specific cutoffs based on the 50 percentile of confidence scores (median) for each organism and tissue (Supplementary Table S2). We specifically used the percentile instead of a fixed cutoff such that we have a comparable number of genes for each tissue and organism irrespective of the differences in the distribution of the scores. Furthermore, we chose exactly the 50 percentile in order to better approximate the expected number of expressed genes in a given tissue (33). For completeness, we also performed the analysis using the 25, 40, 60 and 75 percentiles of confidence scores as cutoffs (see Supplementary Results).

### Orthology-based pathway transferability

KEGG is one of the most well-known and widely used pathway databases (18). It contains manually drawn pathway maps representing molecular interaction and reaction network diagrams. For our analysis, we obtained the set of 216 human KEGG pathways from the STRING v10.5 KEGG benchmark dataset (22). Pathways with less than five genes matched to OGs in either organism were omitted from the analysis, which resulted in a set of 205 human KEGG pathways.

To assess the transferability of each of these pathways from human to another organism, we used the eggNOG mammalian orthology. For each pathway, we mapped each human gene in this pathway to the mOG it belongs to and thereby converted the pathway–gene association to a pathway–OG association. Then, the pathway transferability from human to another organism was calculated as the proportion of pathway genes in the other organism that have orthologs in the same OGs that contain the human genes. This means that a limiting factor of the pathway transfer is the number of mOGs shared between human and the respective organism. In the special case of pathway transferability from human to *all* three analyzed organisms, we only considered the 11 500 mOGs that cover all four organisms.

### Integration of tissue expression data

Given the set of human and orthology-transferred pathways and the tissue expression data from TISSUES, we performed a pathway–tissue analysis, in which we considered for each organism, which pathway genes are expressed in each tissue and compared these among the four analyzed organisms. For each organism, for each tissue and pathway, we calculated the fraction between all pathway genes expressed above the chosen confidence cutoffs (from here on called *expressed pathway genes*) and all genes with any expression information in this pathway (Supplementary Tables S3 and S4). When at least 85% of the orthologous pathway genes with tissue information were above the chosen tissue confidence cutoff, we considered these pathways *expressed* in the given tissue and organism. We chose the cutoff of 85% after inspecting the proportion of expressed genes in several known pathways (*Citrate cycle (TCA cycle)*, *Spliceosome*, *Ribosome*, *Proteasome*, *Oxidative phosphorylation* and *Propanoate metabolism*) such that these pathways were expressed in most tissues (Supplementary Figures S3–S6). Note that there is a connection between this cutoff and the TISSUES confidence cutoff, which we chose for defining whether a gene is expressed in a tissue or not. The fewer genes are considered as expressed, e.g. only the genes in the 75 percentile of confidence scores, the lower we need to put the cutoff for an expressed pathway (for example to 75%) to have an appropriate result (see Supplementary Results and Supplementary Figures S7–S10).

To compare between organisms, we used the eggNOG mOGs that contain at least one orthologous gene for each of the considered organisms. The TISSUES expression confidence scores of two genes from different organisms were considered comparable, if these two genes belong to the same mOG. If several genes from the same organism belong to the same mOG, the highest confidence score was used in the comparison. In addition, we omitted pathways with less than five genes with expression information in either organism, which resulted in 203 pathways suitable for analysis. Note that this number is slightly lower than the 205 transferred pathways due to the restriction of mOGs to have coverage in all four compared organisms.

### Comparison of tissue–pathway combinations across organisms

In order to compare the pathway expression in each tissue between human and the model organisms, we computed the Jaccard index (JI) for each tissue–pathway combination (203 pathways and seven tissues). We defined the JI for a given pathway and tissue between two organisms as the intersection of expressed pathway genes of the two organisms divided by the union of expressed pathway genes in any of the two organisms. A pathway gene is considered expressed if it has a TISSUES confidence score above the chosen organism- and tissue-specific cutoff.

The principal component analysis (PCA) on the JIs for the comparison of human–mouse, human–rat and human–pig was computed using the *scikit-learn* Python package (34) for all pathway–tissue pairs with at least five pathway genes expressed in the given tissue. We used PCA not to reduce the dimensionality but purely as a visualization technique. The loadings for each of the considered variables, which correspond to the JIs for each pair of compared organisms, were computed as the product of the PCA component and the square root of the explained variance for each principal component. Based on the plot of principal components 2 (PC2) and 3 (PC3), we identified a set of pathway–tissue pairs, which are more consistent between human and a specific model organism, by calculating the Euclidean distance of each pathway–tissue pair to the center of the PC2 & PC3 plot. Based on their distance to the loadings, we also grouped the pathway–tissue pairs into six different groups: *mouse, rat, pig, mouse & rat, mouse & pig, rat & pig*. These groups contain pathway–tissue combinations, for which one or two of the model organisms agree more with human than the other(s).

## RESULTS

Several limitations and possibilities about modelling human pathways in animal models arise from publicly available data for the two well-established animal models mouse and rat as well as for the emerging one, pig. The quality of genome assemblies and orthology mapping between organisms have significantly improved in the last years, and increasingly more tissue expression data is becoming available. In contrast, annotations in terms of functions, pathways, and protein interactions are still lacking high-quality experimental data to allow detection of differences between animal models and human. Therefore, we derive animal pathways from curated human pathways using reliable orthology relationships and further integrate these pathways with tissue gene expression data from the animal models. Despite the better coverage and quality of data in mouse and human as compared to rat and pig, we can identify several pathways in specific tissues that agree better with human in one animal model compared to the other two.

### Available functional annotations for animal models are limited compared to human

For the systematic comparison of animal models to human, we need to answer the following important questions: Which data is publicly available and what is the quality of this data? Here, we analyze and compare the following resources: Ensembl for quality of genome assembly and gene annotation (26), eggNOG for orthology relationships (28), text mining of genes and organisms in the biomedical literature, Gene Ontology (GO) functional annotations of the genes (31), the TISSUES database for gene expression (25), and the STRING database of known protein interactions (22). For each of these resources, representative numbers are listed in Table 1 (for further details, see Supplementary Table S1). The amount of data available varies greatly across resources and organisms; for example, mouse is very well covered by most resources, while rat and pig are covered to a lesser extent and their coverage is different for the different resources. Each resource's content and limitations are presented in more detail below.

#### Genome annotations

Based on the overall statistics available from Ensembl release 95 for each of the most recent assemblies, we conclude that there is good annotation of coding genes, while annotation of non-coding RNA genes still needs improvement, especially for rat and pig (Table 1). The corresponding numbers of coding and non-coding genes for human and mouse in GENCODE (27) are very similar to the ones in Ensembl (see Supplementary Table S1). A comprehensive genome quality assessment of human and 20 domesticated animals was performed by Seemann *et al.* (35). At that time, the mouse assembly ranked very high based on its quality as opposed to pig and many of the other animals’ assemblies.

#### eggNOG mammalian orthology

To compare annotations for human, mouse, rat, and pig, we used the eggNOG database 4.5.1, which provides orthologous groups (OGs) at different taxonomic levels. We chose the OGs at mammalian level (mOGs) as they are the most fine-grained OGs that contain all four organisms of interest. The number of genes of each organism that are assigned to mOGs is given in Table 1. The coverage is best for human (86.7%), closely followed by mouse (84.3%) and pig (82.2%), whereas rat has only 76.4%. From the 26 253 mOGs in eggNOG, 11 500 cover all four organisms, e.g. contain at least one gene from each organism, and 8665 of them have one-to-one orthologs for human and the three animal models.

#### Biomedical publications

To approximate the popularity of the three model organisms of interest, we counted how often they are mentioned in PubMed entries (abstracts and publications) using the *tagger* text-mining software (29), which also generates the text-mining associations for the STRING database (23). We considered two different measures: (i) how many PubMed entries mention the organism itself and (ii) how many PubMed entries mention a gene from this organism. The latter number is based on identifying both the organism and the gene in the same PubMed entry and allows us to distinguish publications, which discuss the animal models, especially pig, in connection with veterinary treatments, from the publications, which actually study the molecular biology of the organisms as given by the mentions of genes. As shown in Table 1, mouse and rat are mentioned at least 10 times more often than pig. However, 72% of the entries that mention mouse and only ∼45% for rat and pig appear to specifically study their genes.

#### Gene Ontology annotations

The Gene Ontology is one the most used resources for functional annotation of genes and proteins. GO annotations can be supported by different types of evidence, including experimental, author statements, computationally inferred such as based on phylogeny, as well as non-curated electronic annotation. For each of these categories, we listed the number of annotations for each organism. As for other resources, there is an imbalance between the different types of evidence and the different organisms, human having most annotations with experimental (107 301) or author statement support (48 894). By contrast, even mouse has a huge proportion of annotations inferred computationally (170 033), in addition to many experimentally supported ones (89 360). For rat, and especially for pig, most annotations are supported only by computationally inferred evidence or electronic annotations (Table 1).

#### Protein–protein interactions

To assess the availability of molecular interaction data for each organism, we counted the high-confidence (confidence score ≥ 0.7) experimental protein–protein interaction data in STRING v10.5. The lack of such data in most considered organisms is evident with only ∼900 interactions for rat and ∼1300 for mouse and pig, which is surprising considering how well studied mouse and rat are (e.g. as indicated by their mentions in the literature). In all four organisms, however, many protein–protein interactions can be transferred by orthology from the other organisms in STRING (Table 1, *experimental transferred* interactions) due to the good quality annotation of protein-coding genes.

#### Tissue expression data

An important aspect of studying and comparing animal models is the availability and accessibility of tissue expression data. TISSUES 2.0 integrates evidence on mammalian tissue expression from manually curated literature, proteomics, and transcriptomics screens, and automatic text mining. The numbers in Table 1 clearly demonstrate that only a few large-scale experimental datasets cover several tissues. This is especially the case in pig and rat, which generally have poor coverage in terms of tissue expression data. Having sufficient experimental evidence is also a challenge for less studied tissues in human as shown in Supplementary Table S1 and by Palasca *et al.* (25).

### Annotation similarity between organisms is mainly determined by data availability

Although the available functional annotations for animals are limited compared to human, it is still possible to perform a direct comparison between human and the animal models. Our goal is to assess the extent to which the overlap is driven by data availability, as opposed to evolution. Thus, we determined the pairwise overlap of annotations between human and the three model animals and compared these to the overlap between mouse and rat (see Table 2). Assuming data with good quality and coverage, we would, due to the evolutionary relationship, expect the agreement between mouse and rat to be better than between human and mouse. However, it appears that the difference in data availability between organisms impacts the overlap more than the evolutionary relationship does.

For one-to-one orthology mapping we get comparable numbers for each pair of organisms, reflecting the good annotation quality of protein-coding genes in all four genomes. This is also the case when broadening the orthologous groups to contain one-to-many and many-to-many orthology assignments (*common groups*). Using the mammalian-level orthology assignments to compare between organisms, we further analyzed GO annotations based on experimental and author statement evidence type. We observe by far the highest overlap between human and mouse, reflecting that these are the two most studied organisms. The high similarity of inferred GO terms between mouse and rat can be attributed to database curators annotating GO terms based on sequence similarity to the same experimentally characterized human genes (14,16). Finally, the GO terms in pig come from inferred or electronic annotations (Table 1), which is reflected in the large overlap between human and pig in these categories.

When comparing protein–protein interactions from STRING, we observe that the overlap of *experimental* interactions is more heavily influenced by the availability of data than is the overlap of *experimental transferred* interactions. The small number of overlapping experimental interactions between rat and both human and mouse is in part explained by the fewer experimental rat interactions (Table 1). In the case of *experimental transferred*, we see a good overlap of ∼10 000 interactions for human with mouse and pig as well as between mouse and rat, and a somewhat smaller, but still considerable overlap of 6705 interactions between rat and human.

For the analysis of the tissue expression data, we consider the number of tissues and their coverage by experimental datasets. There are at least 19 tissues covered by *at least one* experimental dataset for each of the pairs human–mouse, human–pig and mouse–rat but only 12 tissues for human–rat, which is consistent with the poor tissue coverage for rat (Table 1). In the case of pig, there is data for 20 of the tissues, however, for 13 of them, the evidence originates only from one experimental dataset. As a result, there are only seven tissues (heart, kidney, liver, nervous system, muscle, lung, spleen) that are covered by *at least two* datasets in all pairs of organisms.

In conclusion, we observe that the extent to which the available annotations overlap between pairs of human and animal models depends more on data availability than on how closely related the organisms are. Given the current data, mouse is better annotated than pig and rat and thus has a better overlap with human than with rat.

### Quantification of orthology-based pathway transfer from human to animal models

Our observations so far provide an estimate of how well human and animal models are covered and agree with each other for individual resources and types of annotations. However, this comparison is limited to individual genes or at most, pairs of genes in the case of STRING interactions. Here, we analyze how consistent pathways can be in terms of their gene content at the organism level.

Most pathway databases focus their annotation efforts on human and thus, even for popular model organisms such as mouse and rat, contain only very few experimentally determined and curated pathway interactions. Instead, they resort to using orthology transfer from human to derive pathways for other organisms. This is the case for popular pathway databases such as Reactome (17) and KEGG (18). Similarly, integrative protein interaction databases such as STRING (23) and IID (24) include orthology transfer of interactions as an information source. However, the exact methodology of pathway transfer differs between databases and even between different organisms within the same database. This can easily cause inconsistencies both between and within the databases, which makes it very difficult to make a meaningful pathway comparison of the organisms.

**Figure 1.**
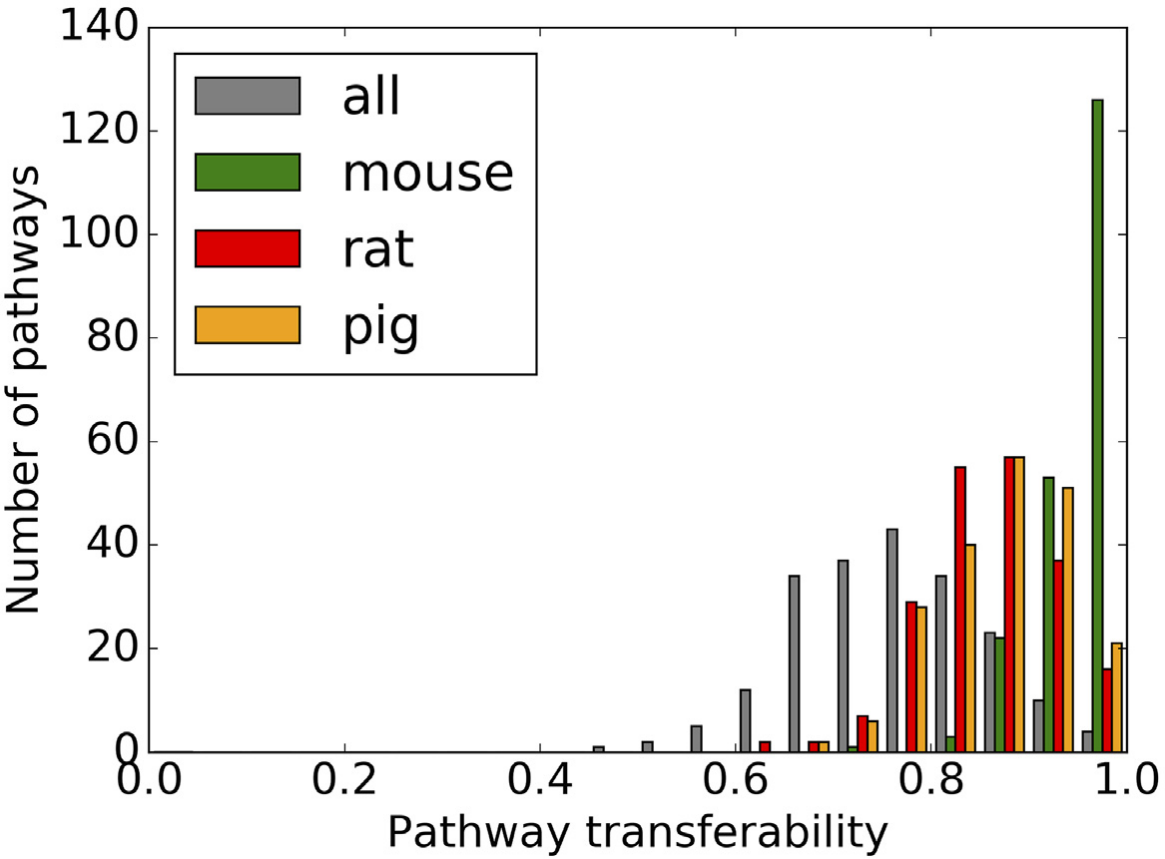
Transferability of 205 KEGG pathways from human to mouse, rat, and pig. Each bar represents the number of pathways, for which a given fraction of genes can be transferred. Transferability to each organism is shown in different colors: mouse in green, rat in red, pig in orange and all organisms in grey.

To meet these challenges, we started with a set of human KEGG pathways and, for each of these pathways, we assessed how well it can be transferred to mouse, rat and pig using the eggNOG orthology relationships within the mammalian taxonomic level. We quantified the *transferability* of each pathway from human to a model organism as the fraction of genes in the human pathway that could be transferred to the model organism in question (Figure 1).

The limiting factor in this comparison is the number of orthologous groups that contain genes from both organisms being compared; this number is lower for rat and pig than for mouse (Table 2). Thus, the pathway transferability from human to mouse has the highest coverage, in terms of both the number of pathways and the number of genes within a pathway. Out of the 205 considered human pathways, 55 can be transferred to mouse completely, compared to only 8 to rat and 12 to pig. The distribution of pathway transferability for mouse has a mean of 95% and ranges between 73% and 100%. In contrast, the distributions of rat and pig are shifted to lower transferability values with a mean of 85% and 87% (minimum of 61% and 65%), respectively.

We are also interested in how well a human pathway can be transferred to all selected animal models at the same time, i.e. what is their overlap (grey colored bars in Figure 1). In this specific case, we consider only orthologous groups that contain at least one gene from each of the four organisms. Overall, there is a good agreement between human and the three animal models with a pathway transferability range between 48% and 100% and a mean of 76%. This range means that for all pathways (but one), more than half of the pathway genes can be transferred from human to mouse, rat, and pig.

The pathways that can be transferred best to all organisms are mainly pathways in the KEGG categories *Metabolism, Replication and repair* or *Human diseases*. A complete list of pathways and number of genes transferred for each of them for each model organism can be found in Supplementary Table S5. For example, the largest pathways among the ones, which are 100% transferable between human and mouse, include *Prostate cancer* (81 genes), *TGF-beta* (67 genes) and *Adipocytokine signaling* pathways (61 genes), for rat they belong to the *Glycan biosynthesis and metabolism* KEGG subcategory (15 pathway genes on average), and for pig they include *RNA polymerase* (29 genes), *Mismatch repair* (22 genes) and *Steroid biosynthesis* (19 genes). The least transferable pathways (50–60% of genes transferred) relate to the nervous system (*Long-term potentiation* and *Dopaminergic synapse*), certain signaling pathways (such as *Notch* and *VEGF signaling* pathways), and the *Circadian rhythm* pathway. The latter is consistent with rat and mouse being nocturnal animals.

### Detection of tissue-specific and broadly-expressed pathways through data integration

In the last couple of years, more and more healthy tissue expression data for animal has been produced and made available in public repositories (36–39). However, this data is difficult to compare across datasets or organisms. Thus, in a previous study, we introduced the TISSUES database (25), which contains tissue expression evidence for human, mouse, rat, and pig in the form of confidence scores that are designed to simplify comparison across datasets and organisms.

In order to explore the differences between the human and animal models at both pathway and tissue level, we integrated the orthology-transferred KEGG pathways with expression data from TISSUES for each organism. We define a pathway gene to be *expressed* in a tissue if it has a confidence score above the chosen cutoff (see Methods for details). In this analysis, we considered the confidence scores based on experimental evidence for seven tissues with good coverage, i.e. at least two experimental datasets available for each organism. We also performed the analysis using the scores that integrate all types of evidence in the TISSUES database as well as different cutoffs for the tissue confidence scores (Supplementary Results and Supplementary Tables S12 and S13).

Out of the 205 pathways, we analyzed only those containing at least five orthologous genes for each of the four compared organisms, resulting in a set of 203 KEGG pathways. Supplementary Table S3 provides the complete list of pathways and, for each of them, the proportion of genes expressed in each tissue and organism. On average in all tissues and organisms, 59.7% of the pathway genes are expressed (Supplementary Table S4). In human, the average across tissues and pathways is 62.1%, while for mouse it is 59.9%, for rat 57.7% and for pig 59.3%. Tissue-wise, we observe that the most pathway genes are expressed in the kidney and liver (>62%), closely followed by lung and spleen with ∼62%, and the least are expressed in the nervous system, heart and muscle (∼57%).

Furthermore, we specifically considered the almost completely *expressed* pathways, which we define as those having at least 85% of the orthologous pathway genes expressed in a specific tissue for each organism (Table 3 and Supplementary Figure S4A). Note that the number of expressed pathways for each tissue and organism is affected both by the requirement of 85% pathway genes as well as the tissue confidence cutoffs, which were chosen such that only the genes with a confidence score above the median value for each tissue were considered expressed. We also performed a more detailed analysis on the connection between these two cutoffs and the robustness of the findings using different cutoffs (see Supplementary Results). Overall, the numbers vary among tissues and organisms, but there are some specific trends. For example, liver has the highest number of expressed pathways (between 29 and 37) in all four organisms, followed by kidney with 26 expressed pathways in human, 25 in mouse and rat, and 19 in pig. For the remaining tissues, we observe a range between 8 and 16 expressed pathways depending on the specific organism and tissue.

**Table 3.** Number of pathways expressed in each tissue and organism

Tissue/organism	Human	Mouse	Rat	Pig
Heart	12	16	14	8
Kidney	26	25	25	19
Liver	**36**	**37**	**29**	**3** **2**
Lung	11	12	13	14
Muscle	12	14	13	12
Nervous system	12	15	10	12
Spleen	12	13	10	13

A pathway is considered expressed if 85% of the pathway genes are above the chosen tissue confidence cutoff. The analysis was done on the 203 human KEGG pathways and the same number of transferred pathways for mouse, rat and pig using the experimental confidence scores from TISSUES for the seven tissues with support by at least two experimental datasets. The highest number of pathways for each organism (each column) is indicated by a bold font.

Based on the number of tissues, in which a pathway is expressed (Supplementary Figure S5A and Supplementary Table S6), we can divide the pathways into broadly expressed and tissue-specific pathways. The *Citrate cycle (TCA cycle)* is an example of a broadly expressed KEGG pathway with >92% of pathway genes expressed in each organism in all seven tissues (except for lung in mouse) as shown in Supplementary Figure S6A. By contrast, the *Axon guidance* KEGG pathway (Supplementary Figure S6B) is – not surprisingly – much more expressed in the nervous system in all organisms (average of 63%) compared to all other tissues (average of 47% over tissues and organisms). Among the 203 pathways, we find 16, 18, 16 and 13 to be expressed in at least three tissues in human, mouse, rat and pig, respectively (Supplementary Table S7). Of these, 10 pathways are expressed in at least three tissues in all four organisms, namely *Citrate cycle (TCA cycle)*, *Spliceosome*, *Ribosome*, *Proteasome*, *Oxidative phosphorylation*, *Protein processing in endoplasmic reticulum*, *Propanoate metabolism*, *Pyruvate metabolism*, *2-Oxocarboxylic acid metabolism* and *Valine, leucine and isoleucine degradation*.

### Evaluation of pathway–tissue agreement between human and animal models

To evaluate which of the pathways expressed in human tissues agree with those in mouse, rat and pig, we assessed how many genes from a pathway are expressed in the same tissue for each pair of organisms (human–mouse, human–rat, human–pig). For each tissue and pathway, we calculated the Jaccard index (JI) as the overlap of expressed pathway genes divided by the union of all expressed pathway genes. A pathway gene is considered expressed if it has a TISSUES confidence score above the chosen cutoff. As a result, for each pathway–tissue combination, we have three JIs of how well this pathway agrees between human and one of the model organisms in the given tissue (Supplementary Table S8).

The average JI over all tissues between human and mouse is 0.63, followed by 0.62 for human–pig, and 0.60 for human–rat (Supplementary Table S9). When we compare the average agreement (over all pathways) between human and the model organisms for each tissue separately, the liver stands out as the tissue with the best agreement for all comparisons, while the remaining tissues are ordered differently depending on the model organism. For example, the tissue with the lowest average JI for the comparisons human–mouse and human-pig is heart (JI of 0.61 and 0.56, respectively), while, for rat, both lung and muscle have the lowest JI (0.57). The distributions of tissue-wise JIs for each of the three comparisons are shown in Supplementary Figure S11 and further confirm that the agreement between the organisms can strongly vary between the tissues.

**Figure 2.**
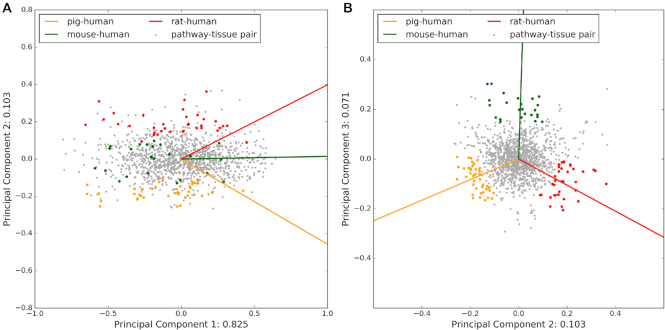
Principal component analysis of the pathway–tissue agreement between human and animal models. PCA was performed on the Jaccard indices (JIs) for all pathway–tissue pairs (grey dots) with at least five expressed pathway genes, where the JIs represent the comparisons human–mouse, human–rat, and human–pig. The PCA loadings are shown as solid lines and colored by the model organism responsible for their direction. While the PC1 & PC2 plot (panel **A**) shows a clear separation between pathway–tissue pairs with high JI and thus, good agreement between human and the respective animal model, the PC2 & PC3 plot (panel **B**) clearly separates the data based on the differences between the animal models. The pathway–tissue pairs located closest to each loading and furthest away from the center of the PC2 & PC3 plot are colored in the same color as the organism loading to indicate that for these pairs, this organism agrees more with human than the others (see Methods section for more details).

To further analyze the similarities and differences between human and the three model organisms on pathway–tissue level, we performed a principal component analysis (PCA) on the JIs for all pathway–tissue pairs, where at least 5 pathway genes are expressed in the given tissue (data shown in Supplementary Table S8). We also plotted the PCA loadings, which show the weight that each model organism has in each principal component (Figure 2). Principal component (PC) 1 accounts for the most variability of the data (82.5%) and highlights the difference between pathway–tissue combinations with high JI and those with low JI, capturing the general agreement between human and all the animal models. From the 34 pathway–tissue combinations that are right-most according to PC1 (PC1 > 0.5), 23 are broadly expressed house-keeping pathways, such as *Citrate cycle* and *Proteasome*. Of those, the highest number of pathways is associated with liver tissue and none of them with the lung. The 43 left-most pathway–tissue pairs according to PC1 (PC1 < –0.5) are mostly small pathways (average size of 8.6 human genes) with low JI (average JI of 0.24 for rat and 0.3 for mouse and pig) and they are distributed across all tissues.

Based on the PCA analysis, PC2 and PC3 separate the three animal models from each other with explained variance of 10.3% and 7.1%, respectively. The loadings of PC2 separate pathway–tissue pairs that show good agreement between human and rat, but poor agreement between human and pig from those showing the opposite behavior. Meanwhile, the loadings of PC3 separate the pathway–tissue pairs based on whether they specifically (or specifically not) show better agreement between human and mouse. We thus used the PC2 & PC3 plot to further identify combinations of pathways and tissues, for which the agreement between human and one of the model organisms is better than with the others. Pathway–tissue pairs that are close to the center of the PC2 & PC3 plot show consistent agreement between human and any of the model organisms; this agreement can be consistently good, if all the JIs are high, or consistently bad, if the JIs are low. In contrast, any point that is very far from the center of the PC2 & PC3 plot represents a specific pathway–tissue combination, for which one of the model organisms has better agreement with human than the others (for a distance distribution see Supplementary Figure S12A). If such a pair is close to one of the three loadings, we assign it to that model organism (mouse, rat, or pig) and consider this pathway–tissue pair to agree more with human and the model organism given by the loading than the other two model organisms (see Supplementary Figure S12B and Methods section). In the cases, where a pathway–tissue combination is between two loadings and furthest away from the third one, we assign it to a category shared between two of the model organisms: mouse & rat (opposite of pig), mouse & pig (opposite of rat), and rat & pig (opposite of mouse). We observed consistent results for the top 100, 200 and 500 pathway–tissue pairs, which are located furthest away from the center (Supplementary Table S10). We observed similar trends when applying the analysis on all data in the TISSUES database (Supplementary Figure S13) and when varying the tissue confidence cutoff (Supplementary Figure S14). Here, we show and discuss only the top 200 in more detail (Table 4).

**Table 4.** Top pathway–tissue combinations showing distinct agreement between human and a model organism

	Mouse	Rat	Pig	Mouse & rat	Rat & pig	Mouse & pig
Pathway–tissue pairs	24	35	41	44	20	36
# pathways	20	28	34	34	17	27
Average JI	0.72	0.74	0.72	0.69	0.62	0.70
# pathways by tissue						
Heart	4	7	5	**10**	1	2
Kidney	3	8	4	8	1	5
Liver	2	3	6	6	2	4
Lung	**7**	2	8	4	4	**12**
Muscle	4	1	**10**	9	1	4
Nerv. system	3	5	2	5	**6**	6
Spleen	1	**9**	6	2	5	3

This table shows the top 200 pairs, while Supplementary Table S10 gives an overview of the top 100 and 500 pathway–tissue pairs. The first three columns indicate the numbers of pairs, for which one of the model organisms (mouse, rat or pig) is specifically more consistent with human than the other two. The last three columns refer to the pairs, which are shared between two model organisms and thus consistent with human in a similar way for both organisms. The average Jaccard index (JI) for all pathway–tissue pairs assigned to a group is also listed. For each tissue row, the number of (#) pathways assigned to this tissue is listed. Numbers shown in bold indicate the tissue covered by the highest number of pathways for each column.

Among the top 200 in the three compared organisms (Supplementary Table S11), pig has the largest number of 41 pathway–tissue pairs, which show agreement with human for this organism only. The 24 pathway–tissue combinations, which are more consistent between mouse and human, have an average JI of 0.72 and cover all analyzed tissues. Specifically, lung tissue has the largest coverage of seven pathways, while spleen has the lowest with one. For the 35 pathway–tissue pairs, for which rat specifically agrees more with human, the average JI is 0.74 and the largest number of nine pathways is associated with spleen, while the lowest of one with muscle. The 41 pairs, which show good agreement specifically between pig and human, have an average JI of 0.72 and also cover the seven analyzed tissues. Whereas only two of these pathways are associated with nervous system, 10 are with muscle.

Half of the top 200 pathway–tissue combinations were not assigned to one specific organism, but instead to two organisms, which show similar, higher consistency with human than the third organism does. Mouse & rat have the highest number of 44 such shared pathway–tissue pairs, with 10 pathways assigned to heart, nine to muscle and only two to spleen. Since mouse and rat are closely related to each other, this result is not surprising. The high number of 36 pathway–tissue pairs, for which mouse & pig are consistent with human, comes as a close second. Out of these, lung has the largest coverage of 12 pathways, while heart and spleen are represented only by two and three pathways, respectively. For the 20 pathway–tissue pairs, for which rat & pig agree with human more than mouse, six are associated with nervous system, and only one pathway is selected for heart, kidney, and muscle.

Overall, the pathway–tissue pairs that show distinct consistency between human and certain model organisms distribute as follows. The two biggest groups are the 41 pathway–tissue pairs unique to pig and the 44 shared by mouse & rat, i.e. not seen in pig. Of these, 19 pathways relate to muscle. For pig, these pathways are in the KEGG category *Organismal Systems*, while for rodents they are in the *Metabolism* category. The next two groups are those unique to rat (35 pathways) and those shared by mouse & pig (36 pathways), i.e. not seen in rat. For the latter, lung stands out by the highest number of 12 pathways. Finally, the liver stands out as the tissue with the most expressed pathways and the one for which all three animal models agree equally well with human.

Although there are limitations due to varying data availability for each organism, our findings indicate that we can successfully approximate the tissue-specific pathway activity and identify similarities and differences between the three considered model organisms and human.

## DISCUSSION

To summarize our observations, there is an abundance of both experimental and inferred information with good quality for human, including genome quality, orthology relationships, biomedical literature, tissue expression data, gene annotations, and protein associations. Unfortunately, the same is not the case for mouse, rat or pig. While mouse is very often mentioned in the literature and is well covered by tissue expression data and GO annotations, there are very few experimentally determined protein associations reported for it. Meanwhile there is a shortage of most types of annotations and data for both rat and pig. Thus, one of the biggest limitations of the current analysis is the availability of public data for model organisms. This can be improved in the future by encouraging researchers to make publicly available more experimental, curated, high-quality findings generated for organisms other than human, especially when these organisms are popular model animals such as mouse and rat.

Pathway transferability, both in our study and in pathway databases in general, is limited by the data availability and agreement of the individual resources, in particular, the amount of pathway annotations and the quality of orthology relationships. Nevertheless, the pathway transfer from human works very well for mouse (95% on average) and fairly well for rat or pig (85% and 87% on average, respectively). The pathway transferability also highlights the extent to which the animal models agree with human at a pathway level given the available data. Due to the lack of organism-specific information on pathways, we are not able to detect more pronounced differences between the organisms by using only this type of data. Ideally, we would like to have one single resource with pathways that are curated separately for each organism. Using it would allow us to identify the specific parts of the pathways that are only present in the animal model but not in human. Unfortunately, this is not possible with the current pathway and interaction databases, even though individual resources such as the Mouse and Rat Genome Database (14,16) try to collect and provide organism-specific data. Therefore, we are in practice forced to think of the human curated pathways as more general representations of what is happening in any tissue. We then try to approximate how these pathways behave in specific tissues or model organisms through integration of other types of data such as tissue expression.

The availability of organism-specific tissue expression datasets is considerably better, although still far from ideal. The deposited datasets often come from one individual and tissue and only sometimes cover several tissues in the same individual(s). This gap has been decreasing lately as more and more high-throughput sequencing data is being generated and deposited in public repositories for human (36–38) and farm animals, including pig (39). For example, several large-scale sequencing and annotation efforts have been undertaken by the FAANG (Functional Annotation of ANimal Genomes) consortium with the goal to improve the functional annotation of animal genomes, including pig, goat, sheep, cattle, horse, and chicken (40). These efforts will improve both the genome quality and gene annotation. However, a limitation is still the need to update resources based on the new genome assemblies, which does not always happen quickly enough. Furthermore, there is a clear need for more resources like the TISSUES database, which can calibrate the data from the different technologies and organisms and make it comparable. This also means that the analysis performed here can be significantly improved in the future once more and better quality data becomes available. Another possibility would be to extend the current analysis to include other less popular model animals.

Another important aspect and possible limitation of our analysis is the comparability between organisms. Most importantly, we need well defined orthology relationships between the compared organisms. Identifying orthology between species has improved over the years (41) and allows us to compare even organisms, which are evolutionarily more distant (42). However, orthology assignments are still heavily influenced by the quality of the underlying genomes and their annotations. In our case this means that some of the orthology relationships between human and pig or human and rat might be missing due to the annotation quality of these genomes at the time when the orthology resources were constructed and updated. This lack of complete orthology relationships influences both the pathway transferability and the extent, to which the organisms agree with each other at pathway–tissue level, and thus only allows us to see part of the whole picture now. However, with better annotated genome assemblies and improved orthology, we expect that our framework will reveal an even more complete picture of the similarities and differences between human and different animal models.

The comparison of individual types of data between the selected four organisms indicates that the observed agreement is more driven by the availability of data than by evolutionary relationships. This is likely due to the lack of organism-specific data at various levels. However, we also showed that through integration of pathway data with tissue expression, we can identify both similarities between human and the model organisms and differences with respect to how well the animal models agree with human at a pathway–tissue level. The resulting pathway–tissue–organism associations revealed both expected and unexpected findings as mentioned previously. For example, so-called house-keeping pathways consist primarily of genes that we see expressed in most tissues and organisms, while other pathways were found to be much more tissue-specific. In terms of pathway–tissue differences between the organisms, all tissues except for the liver were associated with more pathways, for which only one or two, but not all three model organisms were consistent with human. With respect to the question, which of these animal models is best suited for modelling a human disease, we can conclude that there is no universal answer and that it depends on the specific tissue and sometimes even the specific pathways involved in the disease.

To make sure that this specific approach of integrating orthology-derived pathways with tissue expression data from human and animal models is robust with respect to the chosen algorithms and cutoffs, we performed a robustness analysis. In order to use the confidence scores for gene–tissue associations from the TISSUES database, we needed to set a cutoff for whether a gene is expressed or not in a given tissue, which is not a straightforward choice. In addition, when identifying which and how many pathways are expressed in a given tissue, we chose a cutoff for the number of expressed pathway genes. The robustness analysis confirmed that, although the absolute numbers change, the trends remain the same, and thus, our findings are consistent and reproducible irrespective of the specific cutoffs chosen.

Our systematic data integration of pathways with tissue expression enables the investigation of mammalian pathway activity in several different healthy tissues of mouse, rat and pig as well as the comparison with the corresponding human tissues. We highlight tissue-specific features of the pathways and point out similarities and differences between human and the model organisms. Ultimately, we identify distinct pathway–tissue combinations, which are specifically more consistent with human for either of the three studied animal models. These findings can support researchers in the decision of which model organism to choose for a human disease of interest.

In the current analysis, we focused only on the three animal models mouse, rat and pig and on the seven tissues, which are well covered by experimental datasets in the TISSUES database. However, if the used resources become more elaborate in the future, it should be possible to conduct the same type of analysis on more tissues and for more model organisms. This would of course require enough tissue expression data that can be calibrated and made comparable, for example, as done for the TISSUES database. Furthermore, although we based our study on the KEGG pathways database, our workflow for data integration and comparison is applicable to other pathway databases or gene–phenotype and gene–disease associations. The presented framework can also be extended to study the similarity of pathways upon activation or perturbation or to take into account the effect of specific genes, drugs or even diseases on the pathways in the same tissue for different model organisms, given that such a comprehensive collection of data exists and is made publicly available. Thus, future analysis would require the systematic assembly of associations between pathways, tissues and diseases to further aid researchers in choosing the best model organism for studying human diseases.

## DATA AVAILABILITY

This study includes no data deposited in external repositories. All results generated in this study are included as supplementary files.

## Supplementary Material

gkab012_Supplemental_Files

## References

[1] Young R.S., Hayashizaki Y., Andersson R., Sandelin A., Kawaji H., Itoh M., Lassmann T., Carninci P., Consortium F., Bickmore W.A. et al. The frequent evolutionary birth and death of functional promoters in mouse and human. Genome Res. 2015; 25:1546–1557. 26228054 10.1101/gr.190546.115 PMC4579340

[2] Simon M.M., Greenaway S., White J.K., Fuchs H., Gailus-Durner V., Wells S., Sorg T., Wong K., Bedu E., Cartwright E.J. et al. A comparative phenotypic and genomic analysis of C57BL/6J and C57BL/6N mouse strains. Genome Biol. 2013; 14:R82. 23902802 10.1186/gb-2013-14-7-r82 PMC4053787

[3] Pizzollo J., Nielsen W.J., Shibata Y., Safi A., Crawford G.E., Wray G.A., Babbitt C.C. Comparative serum challenges show divergent patterns of gene expression and open chromatin in human and chimpanzee. Genome Biol. Evol. 2018; 10:826–839. 29608722 10.1093/gbe/evy041 PMC5848805

[4] Santpere G., Lopez-Valenzuela M., Petit-Marty N., Navarro A., Espinosa-Parrilla Y. Differences in molecular evolutionary rates among microRNAs in the human and chimpanzee genomes. BMC Genomics. 2016; 17:528. 27474039 10.1186/s12864-016-2863-3 PMC4966751

[5] Aigner B., Allison W.T., Andreatini R., Antonelli M., Arndt S.S., Austin A., Brand C., Bukowska J., Caprariello A.C., Carlisle R.E. et al. Conn P.M. Animal Models for the Study of Human Disease. 2017; Academic Press.

[6] Seok J., Warren H.S., Cuenca A.G., Mindrinos M.N., Baker H.V., Xu W., Richards D.R., McDonald-Smith G.P., Gao H., Hennessy L. et al. Genomic responses in mouse models poorly mimic human inflammatory diseases. Proc. Natl. Acad. Sci. U.S.A. 2013; 110:3507–3512. 23401516 10.1073/pnas.1222878110 PMC3587220

[7] Groop L., Pociot F. Genetics of diabetes - are we missing the genes or the disease. Mol. Cell. Endocrinol. 2014; 382:726–739. 23587769 10.1016/j.mce.2013.04.002

[8] Takao K., Miyakawa T. Genomic responses in mouse models greatly mimic human inflammatory diseases. Proc. Natl. Acad. Sci. 2015; 112:1167–1172. 25092317 10.1073/pnas.1401965111 PMC4313832

[9] Weidner C., Steinfath M., Opitz E., Oelgeschläger M., Schönfelder G. Defining the optimal animal model for translational research using gene set enrichment analysis. EMBO Mol. Med. 2016; 8:831–838. 27311961 10.15252/emmm.201506025 PMC4967938

[10] Nelson D.R., Zeldin D.C., Hoffman S.M.G., Maltais L.J., Wain H.M., Nebert D.W. Comparison of cytochrome P450 (CYP) genes from the mouse and human genomes, including nomenclature recommendations for genes, pseudogenes and alternative-splice variants. Pharmacogenetics. 2004; 14:1–18. 15128046 10.1097/00008571-200401000-00001

[11] Puccinelli E., Gervasi P.G., Longo V. Xenobiotic metabolizing cytochrome P450 in pig, a promising animal model. Curr. Drug Metab. 2011; 12:507–525. 21476973 10.2174/138920011795713698

[12] Hu J.X., Thomas C.E., Brunak S. Network biology concepts in complex disease comorbidities. Nat. Rev. Genet. 2016; 17:615–629. 27498692 10.1038/nrg.2016.87

[13] Kitsak M., Sharma A., Menche J., Guney E., Ghiassian S.D., Loscalzo J., Barabási A.-L. Tissue specificity of human disease module. Sci. Rep. 2016; 6:35241. 27748412 10.1038/srep35241 PMC5066219

[14] Bult C.J., Blake J.A., Smith C.L., Kadin J.A., Richardson J.E. Mouse Genome Database Group Mouse Genome Database (MGD) 2019. Nucleic Acids Res. 2019; 47:D801–D806. 30407599 10.1093/nar/gky1056 PMC6323923

[15] Groenen M.A.M., Archibald A.L., Uenishi H., Tuggle C.K., Takeuchi Y., Rothschild M.F., Rogel-Gaillard C., Park C., Milan D., Megens H.-J. et al. Analyses of pig genomes provide insight into porcine demography and evolution. Nature. 2012; 491:393–398. 23151582 10.1038/nature11622 PMC3566564

[16] Smith J.R., Hayman G.T., Wang S.-J., Laulederkind S.J.F., Hoffman M.J., Kaldunski M.L., Tutaj M., Thota J., Nalabolu H.S., Ellanki S.L.R. et al. The year of the rat: the rat genome database at 20: a multi-species knowledgebase and analysis platform. Nucleic Acids Res. 2020; 48:D731–D742. 31713623 10.1093/nar/gkz1041 PMC7145519

[17] Fabregat A., Jupe S., Matthews L., Sidiropoulos K., Gillespie M., Garapati P., Haw R., Jassal B., Korninger F., May B. et al. The Reactome Pathway Knowledgebase. Nucleic Acids Res. 2018; 46:D649–D655. 29145629 10.1093/nar/gkx1132 PMC5753187

[18] Kanehisa M., Furumichi M., Tanabe M., Sato Y., Morishima K. KEGG: new perspectives on genomes, pathways, diseases and drugs. Nucleic Acids Res. 2017; 45:D353–D361. 27899662 10.1093/nar/gkw1092 PMC5210567

[19] Licata L., Briganti L., Peluso D., Perfetto L., Iannuccelli M., Galeota E., Sacco F., Palma A., Nardozza A.P., Santonico E. et al. MINT, the molecular interaction database: 2012 update. Nucleic Acids Res. 2012; 40:D857–D861. 22096227 10.1093/nar/gkr930 PMC3244991

[20] Kerrien S., Aranda B., Breuza L., Bridge A., Broackes-Carter F., Chen C., Duesbury M., Dumousseau M., Feuermann M., Hinz U. et al. The IntAct molecular interaction database in 2012. Nucleic Acids Res. 2012; 40:D841–D846. 22121220 10.1093/nar/gkr1088 PMC3245075

[21] Oughtred R., Stark C., Breitkreutz B.-J., Rust J., Boucher L., Chang C., Kolas N., O’Donnell L., Leung G., McAdam R. et al. The BioGRID interaction database: 2019 update. Nucleic Acids Res. 2019; 47:D529–D541. 30476227 10.1093/nar/gky1079 PMC6324058

[22] Szklarczyk D., Morris J.H., Cook H., Kuhn M., Wyder S., Simonovic M., Santos A., Doncheva N.T., Roth A., Bork P. et al. The STRING database in 2017: quality-controlled protein-protein association networks, made broadly accessible. Nucleic Acids Res. 2017; 45:D362–D368. 27924014 10.1093/nar/gkw937 PMC5210637

[23] Szklarczyk D., Gable A.L., Lyon D., Junge A., Wyder S., Huerta-Cepas J., Simonovic M., Doncheva N.T., Morris J.H., Bork P. et al. STRING v11: protein-protein association networks with increased coverage, supporting functional discovery in genome-wide experimental datasets. Nucleic Acids Res. 2019; 47:D607–D613. 30476243 10.1093/nar/gky1131 PMC6323986

[24] Kotlyar M., Pastrello C., Malik Z., Jurisica I. IID 2018 update: context-specific physical protein-protein interactions in human, model organisms and domesticated species. Nucleic Acids Res. 2019; 47:D581–D589. 30407591 10.1093/nar/gky1037 PMC6323934

[25] Palasca O., Santos A., Stolte C., Gorodkin J., Jensen L.J. TISSUES 2.0: an integrative web resource on mammalian tissue expression. Database J. Biol. Databases Curation. 2018; 2018:bay003. 10.1093/database/bay003 PMC5808782 29617745

[26] Zerbino D.R., Achuthan P., Akanni W., Amode M.R., Barrell D., Bhai J., Billis K., Cummins C., Gall A., Girón C.G. et al. Ensembl 2018. Nucleic Acids Res. 2018; 46:D754–D761. 29155950 10.1093/nar/gkx1098 PMC5753206

[27] Frankish A., Diekhans M., Ferreira A.-M., Johnson R., Jungreis I., Loveland J., Mudge J.M., Sisu C., Wright J., Armstrong J. et al. GENCODE reference annotation for the human and mouse genomes. Nucleic Acids Res. 2019; 47:D766–D773. 30357393 10.1093/nar/gky955 PMC6323946

[28] Huerta-Cepas J., Szklarczyk D., Forslund K., Cook H., Heller D., Walter M.C., Rattei T., Mende D.R., Sunagawa S., Kuhn M. et al. eggNOG 4.5: a hierarchical orthology framework with improved functional annotations for eukaryotic, prokaryotic and viral sequences. Nucleic Acids Res. 2016; 44:D286–D293. 26582926 10.1093/nar/gkv1248 PMC4702882

[29] Pafilis E., Frankild S.P., Fanini L., Faulwetter S., Pavloudi C., Vasileiadou A., Arvanitidis C., Jensen L.J. The SPECIES and ORGANISMS resources for fast and accurate identification of taxonomic names in text. PLoS One. 2013; 8:e65390. 23823062 10.1371/journal.pone.0065390 PMC3688812

[30] Doncheva N.T., Jensen L.J. Text mining results for gene and organism mentions. 2020; 10.6084/M9.FIGSHARE.13266548.V1.

[31] The Gene Ontology Consortium. Expansion of the Gene Ontology knowledgebase and resources. Nucleic Acids Res. 2017; 45:D331–D338. 27899567 10.1093/nar/gkw1108 PMC5210579

[32] Gremse M., Chang A., Schomburg I., Grote A., Scheer M., Ebeling C., Schomburg D. The BRENDA Tissue Ontology (BTO): the first all-integrating ontology of all organisms for enzyme sources. Nucleic Acids Res. 2011; 39:D507–D513. 21030441 10.1093/nar/gkq968 PMC3013802

[33] Islam S., Zeisel A., Joost S., La Manno G., Zajac P., Kasper M., Lönnerberg P., Linnarsson S. Quantitative single-cell RNA-seq with unique molecular identifiers. Nat. Methods. 2014; 11:163–166. 24363023 10.1038/nmeth.2772

[34] Pedregosa F., Varoquaux G., Gramfort A., Michel V., Thirion B., Grisel O., Blondel M., Prettenhofer P., Weiss R., Dubourg V. et al. Scikit-learn: machine learning in Python. J. Mach. Learn. Res. 2011; 12:2825–2830.

[35] Seemann S.E., Anthon C., Palasca O., Gorodkin J. Quality assessment of domesticated animal genome assemblies. Bioinforma. Biol. Insights. 2015; 9:49–58. 10.4137/BBI.S29333 PMC4898645 27279738

[36] Barrett T., Wilhite S.E., Ledoux P., Evangelista C., Kim I.F., Tomashevsky M., Marshall K.A., Phillippy K.H., Sherman P.M., Holko M. et al. NCBI GEO: archive for functional genomics data sets–update. Nucleic Acids Res. 2013; 41:D991–D995. 23193258 10.1093/nar/gks1193 PMC3531084

[37] Wu C., Orozco C., Boyer J., Leglise M., Goodale J., Batalov S., Hodge C.L., Haase J., Janes J., Huss J.W. et al. BioGPS: an extensible and customizable portal for querying and organizing gene annotation resources. Genome Biol. 2009; 10:R130. 19919682 10.1186/gb-2009-10-11-r130 PMC3091323

[38] Brazma A., Parkinson H., Sarkans U., Shojatalab M., Vilo J., Abeygunawardena N., Holloway E., Kapushesky M., Kemmeren P., Lara G.G. et al. ArrayExpress–a public repository for microarray gene expression data at the EBI. Nucleic Acids Res. 2003; 31:68–71. 12519949 10.1093/nar/gkg091 PMC165538

[39] Bastian F., Parmentier G., Roux J., Moretti S., Laudet V., Robinson-Rechavi M. Bgee: Integrating and comparing heterogeneous transcriptome data among species. Data Integr. Life Sci. 2008; 5109:124–131.

[40] Giuffra E., Tuggle C.K. FAANG Consortium Functional Annotation of Animal Genomes (FAANG): current achievements and roadmap. Annu. Rev. Anim. Biosci. 2019; 7:65–88. 30427726 10.1146/annurev-animal-020518-114913

[41] Altenhoff A.M., Boeckmann B., Capella-Gutierrez S., Dalquen D.A., DeLuca T., Forslund K., Huerta-Cepas J., Linard B., Pereira C., Pryszcz L.P. et al. Standardized benchmarking in the quest for orthologs. Nat. Methods. 2016; 13:425–430. 27043882 10.1038/nmeth.3830 PMC4827703

[42] Huerta-Cepas J., Szklarczyk D., Heller D., Hernández-Plaza A., Forslund S.K., Cook H., Mende D.R., Letunic I., Rattei T., Jensen L.J. et al. eggNOG 5.0: a hierarchical, functionally and phylogenetically annotated orthology resource based on 5090 organisms and 2502 viruses. Nucleic Acids Res. 2019; 47:D309–D314. 30418610 10.1093/nar/gky1085 PMC6324079

