# Supplementary material for: Human pathways in animal models: possibilities and limitations
Source: Nucleic Acids Res. 2021 Feb 1;49(4):1859–71. doi: 10.1093/nar/gkab012 (PMC7913694; doi:10.1093/nar/gkab012)
Supplement: gkab012_Supplemental_Files [file gkab012_supplemental_files.zip › Suppl_Results_and_Figures_revised.pdf]

Human pathways in animal models: possibilities and limitations
Supplemental materials

Nadezhda T. Doncheva[1,2,3], Oana Palasca[1,2,3], Reza Yarani[4], Thomas Litman[5,6], Christian Anthon[1,2], Martien A. M. Groenen[7], Peter F. Stadler[1,8,9,10,11,12], Flemming Pociot[1,4,13], Lars J. Jensen[1,3,*], Jan Gorodkin[1,2,*]

[1]Center for non-coding RNA in Technology and Health, University of Copenhagen, 1871 Frederiksberg, Denmark
[2]Department of Veterinary and Animal Sciences, University of Copenhagen, 1870 Frederiksberg, Denmark
[3]Novo Nordisk Foundation Center for Protein Research, University of Copenhagen, 2200 Copenhagen, Denmark
[4]Translational Type 1 Diabetes Research, Steno Diabetes Center Copenhagen, 2820 Gentofte, Denmark
[5]Department of Immunology and Microbiology, University of Copenhagen, 2200 Copenhagen, Denmark
[6]Exploratory Biology, LEO Pharma A/S, 2750 Ballerup, Denmark
[7]Animal Breeding and Genomics, Wageningen University & Research, 6700 Wageningen The Netherlands
[8]Bioinformatics Group, Department of Computer Science; Interdisciplinary Center for Bioinformatics; German Centre for Integrative Biodiversity Research (iDiv) Halle-Jena-Leipzig; Competence Center for Scalable Data Services and Solutions Dresden-Leipzig; Leipzig Research Center for Civilization Diseases; and Centre for Biotechnology and Biomedicine, University of Leipzig, 04107 Leipzig, Germany
[9]Max Planck Institute for Mathematics in the Sciences, 04103 Leipzig, Germany
[10]Institute for Theoretical Chemistry, University of Vienna, 1090 Vienna, Austria
[11]Facultad de Ciencias, Universidad Nacional de Colombia, Bogotá D.C., Colombia
[12]The Santa Fe Institute, 87501 Santa Fe, NM, United States
[13]Faculty of Health and Medical Sciences, University of Copenhagen, 2200 Copenhagen, Denmark

* To whom correspondence should be addressed: Jan Gorodkin, Tel: +45 35 333578, Email: gorodkin@rth.dk, Lars Juhl Jensen, Tel: +45 35 325025, Email: lars.juhl.jensen@cpr.ku.dk

Supplemental results and discussion

In order to explore the differences between the human and animal models at both pathway and tissue level, we integrated the orthology-transferred KEGG pathways for each organism with expression data from the TISSUES database (1). For the expression data, we considered two different cases: 1) only the experimental evidence scores and 2) the scores integrating all types of evidence included in the database. As part of the analysis, we need to set two different cutoffs to define a) whether a gene is expressed in a given tissue and organism and b) whether a pathway consisting of several genes is considered expressed in a tissue and organism. While we focus only on the experimental data and one set of cutoffs in the main text, here we describe the results using all types of evidence as well as different cutoffs with the experimental evidence scores.

Analysis including all types of tissue evidence

Here we analyzed the seven tissues with good coverage, i.e. at least two experimental datasets available for each organism, and only those pathways containing at least five orthologous genes for each of the four compared organisms, resulting in a set of 203 KEGG pathways. In contrast to the analysis presented in the main text, we used all types of evidence provided by TISSUES, including gene–tissue associations from text-mining, curated knowledge, and experimental datasets. We define a gene to be expressed if it has a confidence score above the 50 percentile (median) of all confidence scores (see Suppl. Table 12, *Tissue confidence cutoffs*). On average in all tissues and organisms, 66.3% of the pathway genes are expressed (Suppl. Table 12, *Proportion of expressed genes*). In human, the average across tissues and pathways is 72.4%, while for mouse it is 66.6%, for rat 65.2% and for pig 61.2%. If we average over all pathways in the four organisms, we observe that the largest percentage of pathway genes are expressed in kidney and liver (~69%), closely followed by lung, heart and nervous system with ~66%, and the lowest percentage are expressed in muscle (~65%) and spleen (~63%). These numbers are higher than the corresponding numbers of the analysis using only experimental data as can be expected when combining different types of evidence.

Then, we considered the “almost completely expressed” pathways, which we define as those having at least 85% of the orthologous pathway genes expressed in a specific tissue for each organism (Suppl. Figure 4B and Suppl. Table 12, *Number of tissues per pathway*). As expected and due to the better data coverage of human, we observe that the number of human pathways expressed in all tissues is higher than for any of the other organisms (also higher compared to using only experimental data as done in Suppl. Figure 4A). For example, for liver and kidney we observe as many as 65 and 64 human pathways expressed, respectively. Liver is also the tissue with the most expressed pathways in the three animal models (47 for mouse, 37 for rat, and 36 for pig). The least expressed pathways for human are in the lung (36 pathways), while for pig there are only 8 in heart. For mouse, the least represented tissue is also the lung with 21 pathways, while for rat there are 16 pathways expressed in the lung and spleen.

In the same way as we did for the experimental data only, we counted the number of tissues in which a pathway is expressed (Suppl. Figure 5B). We observe a similar distribution with most pathways being

expressed in none or only one tissue and a few expressed in all tissues. However, there are many more pathways expressed in more tissues in human than in the other organisms when using all evidence from TISSUES (Suppl. Figure 5B) as compared to using only the experimental one (Suppl. Figure 5A). Both the broadly expressed *Citrate cycle (TCA cycle)* (Suppl. Figure 6C) and the tissue-specific *Axon guidance* KEGG pathway (Suppl. Figure 6D) show a similar trend as in the analysis using only experimental data – one of them is broadly expressed and one is specific to the nervous tissue. Interestingly, the same 10 pathways are expressed in at least three tissues in all four organisms irrespective of the evidence used for the tissue expression data. These are *Citrate cycle (TCA cycle)*, *Spliceosome*, *Ribosome*, *Proteasome*, *Oxidative phosphorylation*, *Protein processing in endoplasmic reticulum*, *Propanoate metabolism*, *Pyruvate metabolism*, *2-Oxocarboxylic acid metabolism,* and *Valine, leucine and isoleucine degradation*. Of the 203 pathways, 57 are expressed in at least three tissues for human and 22 for mouse. For rat, there are 20 expressed pathways in at least three tissues, while for pig there are 13.

To evaluate which of the expressed pathways in human tissues agree with those in pig, mouse, and rat tissues, we assessed how many genes from each pathway are expressed in the same tissue for each pair of organisms (human–mouse, human–rat, human–pig). Then, for each tissue and pathway, we calculated the Jaccard index (JI) as the overlap of expressed pathway genes divided by the union of all expressed pathway genes. As a result, we have three JIs of how well a pathway agrees between human and one of the model organisms in a given tissue (Suppl. Table 12, *Pathway–tissue comparison*). The average JI over all tissues between human and mouse is 0.69, followed by 0.65 for rat and 0.61 for pig. If we compare the agreement between human and the model organisms for each tissue separately, the liver stands out as the tissue with the best agreement for two of the three comparisons (0.74 for mouse–human and 0.65 for pig–human). For rat–human this is the tissue with the second best agreement of 0.67, while nervous system has slightly better JI value of 0.68. The average JIs of the remaining tissues are ordered differently depending on the compared organisms. For example, the tissue with the lowest average JIs for the comparison between human and mouse is nervous system (0.65), for rat it is lung (0.60), and for pig it is heart (0.56).

To further analyze the similarities and differences between human and the three model organisms on pathway–tissue level, we performed a principal component analysis (PCA) on the JIs for all pathway–tissue pairs (Suppl. Figure 13). The PCA looks similar to the one for experimental data only with PC1 separating the pathway–tissues combinations based on their JI, i.e., pairs with high JI are on the right side and those with low JI are on the left side (Suppl. Figure 13A). PC2 and PC3 separate the animal models from each other (Suppl. Figure 13B). We performed the same analysis of using the PCA loadings and the distance to the PC2 & PC3 plot center (Suppl. Figure 13C) to assign the furthest located pathway–tissue pairs to a group, for which one or two of the model organisms agree more with human than the other(s). Although we discuss only the top 200 pathway–tissues pairs, which are most distant from the center of the PCA plot in more detail, the results for the top 100 and 500 are overall consistent with the top 200 (Suppl. Table 12, *Top pathway–tissue pairs*).

The 26 pathway–tissue combinations, which are more similar between mouse and human, have an average JI of 0.83 and cover all analyzed tissues. Specifically, heart tissue has the largest coverage of

9 pathways while nervous system and spleen have the lowest with 1. For the 25 pathway–tissue pairs, for which rat specifically agrees more with human, the average JI is 0.79 and only 6 tissues are represented. The largest number of 14 pathways is associated with the nervous system, while the lowest of 1 with heart and none with lung. The 37 pairs, which show good agreement specifically between pig and human, have a lower average JI of 0.67 in contrast to mouse and rat and cover only 5 out of the 7 analyzed tissues. While no pathways are associated with heart and kidney, 15 of the pathways are specific in lung.

More than half of the top 200 pathway–tissue combinations were assigned to one of the groups with two organisms that are similarly consistent with human: mouse & rat, mouse & pig, and rat & pig. The group of mouse & rat has the highest number of 52 common pairs, from which 17 pathways are assigned to muscle but only 2 to spleen and 3 to nervous system. Since mouse and rat are closely related to each other, it is not surprising that the largest group of pathway–tissue combinations is shared between them. The next group contains 36 pathway–tissue pairs shared between rat & pig, while the last one includes 24 and they are shared between mouse & pig. From the pathways that rat & pig have in common with human, 16 are associated with nervous system, while only 1 with liver. This is consistent with the high number of pathways specifically agreeing more between rat and human in nervous system. Out of the pairs similar between mouse & pig with human (24), muscle is not represented at all, lung and liver have the largest coverage of 8 pathways, and all other tissues are represented by 2 pathways. Even more clearly than for the individual organisms, we observe that some tissues are much more consistent with human for a subset of model organisms and less consistent with the remaining ones (e.g. nervous system, lung and liver).

For example, nervous system is the tissue with the most consistent pathways between human and rat as well as between human and rat & pig considered as a group. Since nervous system has the highest TISSUES confidence scores for human and mouse and lower ones for rat and pig (Suppl. Figures 1-2), we can easily exclude lack of data as a possible reason for this result, thus suggesting that rat and pig are indeed better choices for models of nervous system than mouse. Another interesting and less expected observation is the lack of pathways expressed consistently between pig and human hearts. Although pig hearts are known to be anatomically more similar to human hearts than mouse hearts are, there is evidence for anatomical and physiological differences between all three organisms (2). It is not clear yet how these differences might or not affect the molecular mechanisms and pathways in the heart (3).

Overall, we observe both similarities and differences in the analysis using only experimental data and all types of data from the TISSUES database. One of the big differences is that all data includes manual annotations from UniProt, which are available to a greater extent for human and mouse than for rat and pig, as well as text mining, which is also partly biased towards more studied organisms. Nevertheless, liver is the tissue with the most expressed pathways and least specific differences among model organisms irrespective of the used data. There are also similar numbers of pathway–tissue combinations, for which a specific model organism agrees more with human than the others, but not all tissues are equally covered by the pathways in each of these cases. We also observe more common pathway–tissue pairs for mouse & pig (mostly in lung) than for rat & pig (mostly in nervous system)

using only experimental data. The tissues with the most pathways are the same when using all data, but there are more pairs assigned to the group of rat & pig. Last but not least, the average JIs for each group differ based on which data is used – they are higher for mouse, rat, and mouse & rat using all data and for pig and mouse & pig when using only experimental data.

Robustness analysis using different cutoffs

We performed a robustness analysis using different cutoffs than the ones described in the main text. There are two cutoffs used in the analysis that can influence the results. First, a cutoff that defines which genes are considered expressed in a tissue based on the confidence scores provided by TISSUES. For this, we used as *default* a 50 percentile cutoff based on the genes with any tissue confidence score and further tested the 25, 40, 60, and 75 percentiles. Second, a cutoff that defines when a pathway is considered expressed based on the expression of its member genes. In the main manuscript, we required at least 85% of the genes in a pathway to be expressed. Here, we also considered pathways as expressed if at least 75%, 80%, 90%, or 95% of their genes were expressed.

Varying the cutoff for which genes are expressed in a tissue in the range between the 25 and 75 percentile influences the absolute numbers of genes considered for the analysis (Suppl. Table 13, *Tissue cutoffs 25-75 percentile*). With a cutoff as low as the 25 percentile, there are up to ~12,000 genes expressed in some human tissues (on average ~9,700 in any tissue or organism), while with a cutoff as high as the 75 percentile, there are on average ~3,200 genes expressed in any tissue or organism. If we specifically look at the average number of expressed pathway genes in each tissue and organism (Suppl. Table 13, *Tissue cutoffs avg. pathway expr*), they vary between 81% for the 25 percentile, 59.7% for the 50 percentile, and 36.5% for the 75 percentile. These numbers are as can be expected given the absolute numbers of genes considered to be expressed in each tissue for these cutoffs. An example for the broadly expressed KEGG pathway *Citrate cycle (TCA cycle)* is shown in Suppl. Figure 7. While the pathway can be considered expressed in all tissues and organisms using the *default* 50 percentile confidence cutoff and 85% of expressed pathway genes (Suppl. Figure 3A), this is not the case when we choose a tissue cutoff of 60 or 75 percentile and keep the same cutoff for the expressed pathway genes (Suppl. Figure 7C-D).

This trend is even more clear for the distribution of expressed pathways per tissue and the number of tissues with expressed pathways (Suppl. Figure 8 and 9 and Suppl. Table 13, *Cutoffs #pathways #tissues*), where the 25 and 75 percentile cutoffs are the two extremes with too many or too few pathways expressed in the individual tissues. However, this is only the case when we keep the fixed cutoff of 85% expressed pathway genes to consider a pathway expressed. If we change that to accommodate the lower or higher number of expected expressed genes given the different percentile cutoffs on the tissue confidence (e.g. 95% expressed pathway genes for the 25 percentile and 75% expressed pathway genes for the 75 percentile), the resulting distributions (Supp. Figure 8C,F and 9C,F) look very similar to the ones using the 50 percentile tissue cutoff and the 85% expressed pathway genes cutoff (Suppl. Figure 4A and 5A). For completeness, Suppl. Figure 10 shows the number of expressed pathways per tissue and organism as well as the number of tissues with expressed pathways for the *default* cutoff of 50 percentile and varying cutoffs for the number of expressed pathway genes

(75%, 80%, and 90%). Altogether we can confirm the previous observations that the tissue and pathway cutoff depend on each other and that choosing an appropriate combination of these two cutoffs is important. In addition, the trends observed for liver and kidney to be the tissues with the most expressed pathways (Table 3) are consistent with different cutoffs for the number of expressed pathway genes (Suppl. Table 13, *Pathway cutoffs 75%-95% genes*).

The remaining analyses are not influenced by the expressed pathway cutoff, but only by the tissue confidence cutoffs. For each organism comparison mouse–human, rat–human and pig–human, we calculated their pathway similarity for a given tissue by using the Jaccard index (JI) of the overlap of expressed genes in the one and the other organism. The average JIs over all pathways indicate that the tissue confidence cutoffs influence the similarity between the compared organisms as follows (Suppl. Table 13, *Tissue cutoffs avg. JIs*). The higher the number of expressed genes, the higher the average JI for the similarity of two organisms (0.75 for 25 percentile and 0.49 for the 75 percentile). While liver remains the tissue with the highest JI for all three comparisons with the 50, 60, and 75 percentile cutoffs, it is only among the highest ones for the other two cutoffs (25 and 40), where the highest JI is different for each comparison, e.g. nervous system for human–mouse and lung for rat–human and pig–human. Given a too high or too low number of considered genes, the JI loses its power and is not the right measure for such a comparison.

Our final analysis consists of using PCA on the JIs between the organisms to more easily visualize the trends and pick some interesting pathway–tissue combinations for each model organism. The PCA plots using different percentile cutoffs (25, 40, 60, and 75) appear to be robust and remain very similar to the one using the 50 percentile (Suppl. Figure 14 and Figure 2). While PC1 still captures the difference between good agreement and bad agreement, PC2 and PC3 capture what is different in each organism when compared to human. The only difference between the PCA plots appears to be the rotation of the plot for the different cutoffs. Furthermore, the top 200 pathway–tissue combinations, which show the most distinct differences between the model organisms, consist of a similar number of pairs for each of the six groups: mouse, rat, pig, mouse & rat, mouse & pig, rat & pig (Suppl. Table 13, *Tissue cutoffs top 200*). As for the results with 50 percentile, there are more pairs for the separate groups of pig and rat as compared to the mouse ones, but the joint group of pig & rat has much less pairs than the other two joint groups (mouse & rat and mouse & pig). The most represented tissues and the specific pathway–tissue pairs overlap to a certain degree for the different cutoffs, but are not identical as can be expected due to the different set of genes considered to be expressed in each tissue as well as the limitations of comparing two lists with a fixed cutoff instead of comparing the whole lists.

Overall, the absolute numbers change, but the trends remain similar, showing the robustness of our analyses and conclusions. As expected, more stringent cutoffs decrease the absolute numbers of genes and pathways expressed in a tissue, while less stringent cutoffs increase these numbers. Also, using an inappropriate combination of cutoffs leads to very poor results, i.e. one cannot expect to identify many expressed pathways in a tissue, if more than 95% of the pathway genes are expected to be expressed, but only genes with a tissue confidence score above the 75 percentile are considered expressed in the tissue. Similarly, a too low cutoff for the confidence scores and for the expressed pathway genes leads to results indicating that most pathways are expressed in most tissues.

References

1. Palasca,O., Santos,A., Stolte,C., Gorodkin,J. and Jensen,L.J. (2018) TISSUES 2.0: an integrative web resource on mammalian tissue expression. *Database J. Biol. Databases Curation*, **2018**, bay003.

2. Crick,S.J., Sheppard,M.N., Ho,S.Y., Gebstein,L. and Anderson,R.H. (1998) Anatomy of the pig heart: comparisons with normal human cardiac structure. *J. Anat.*, **193 (Pt 1)**, 105–119.

3. Federspiel,J.D., Tandon,P., Wilczewski,C.M., Wasson,L., Herring,L.E., Venkatesh,S.S., Cristea,I.M. and Conlon,F.L. (2019) Conservation and divergence of protein pathways in the vertebrate heart. *PLoS Biol.*, **17**, e3000437.

List of supplemental figures

Suppl. Figure 1: Distribution of confidence scores per organism for experimental data in TISSUES.

Suppl. Figure 2: Distribution of confidence scores per organism for all data in TISSUES.

Suppl. Figure 3: Proportion of pathway genes expressed in each tissue for broadly expressed pathways.

Suppl. Figure 4: Number of pathways expressed in each tissue using the chosen cutoffs.

Suppl. Figure 5: Number of tissues with expressed pathways using the chosen cutoffs.

Suppl. Figure 6: Proportion of pathway genes expressed in each tissue for two selected pathways.

Suppl. Figure 7: Proportion of pathway genes expressed in the KEGG pathway TCA cycle using different tissue confidence cutoffs.

Suppl. Figure 8: Number of pathways expressed in each tissue using different cutoffs.

Suppl. Figure 9: Number of tissues with expressed pathways using different cutoffs.

Suppl. Figure 10: Number of pathways and tissues using different pathway cutoffs.

Suppl. Figure 11: Distribution of Jaccard indices for all pathways across all tissues.

Suppl. Figure 12: Selection of pathway–tissue pairs based on PC2 and PC3.

Suppl. Figure 13: Principal component analysis of the pathway–tissue agreement between human and animal models for all data in TISSUES.

Suppl. Figure 14: Principal component analysis of the pathway–tissue agreement between human and animal models for different tissue confidence cutoffs.

List of supplemental tables

Suppl. Table 1: Full table of data availability per resource for human, mouse, rat, and pig.

Suppl. Table 2: Tissue confidence cutoffs and number of genes per tissue for experimental data.

Suppl. Table 3: Proportion of expressed pathway genes in each tissue. Proportions are based on the experimental data from the TISSUES database and are provided for each pathway, tissue and organism: human (9606), mouse (10090), rat (10116), and pig (9823).

Suppl. Table 4: Average percentage of pathway genes expressed in each tissue and organism. Percentages are based on the experimental data from the TISSUES database and are calculated from the data in Suppl. Table 3.

Suppl. Table 5: Pathway size and transferability from human to mouse, rat, and pig. Pathway size is the number (#) of orthologous genes for each pathway and pathway transferability is the proportion of transferred (orthologous) genes.

Suppl. Table 6: Number of tissues per organism, in which the pathway is expressed. A pathway is considered expressed in a tissue if 85% of the pathway genes with any experimental tissue information are above the 50 percentile tissue confidence cutoff.

Suppl. Table 7: Number of pathways expressed per organism and tissue. A pathway is considered expressed in a tissue if 85% of the pathway genes with any experimental tissue information are above the 50 percentile tissue confidence cutoff.

Suppl. Table 8: Pathway and tissue agreement between human and the compared organisms. Agreement is based on the experimental data from the TISSUES database and is measured by Jaccard index (JI) for each pair human–mouse, human–rat, and human–pig. For each pathway, the number of expressed genes and proportion of expressed genes compared to all genes with tissue information are given. The values from the PCA analysis (PC1, PC2, PC3, distance from the PC2 & PC3 center) as well as the categories for the top 200 are also included.

Suppl. Table 9: Average Jaccard indices for the agreement between human and the compared organisms. Only experimental data from the TISSUES database is used in the analysis.

Suppl. Table 10: Summary of top 100, 200 and 500 pathway–tissue combinations. In each column, the numbers are given for one or two of the model organisms that are specifically more consistent with human than the other(s). The analysis is based on the experimental data from the TISSUES database.

Suppl. Table 11: Top 200 pathway–tissue combinations in detail. For each organism (mouse, rat, pig) or organism pair (mouse & rat, mouse & pig, rat & pig), the identified, more distinct pathways are listed together with their relevant information.

Suppl. Table 12: Results using all evidence types in TISSUES. This table contains 1) tissue confidence cutoffs and number of genes per tissue and organism; 2) proportion of pathway genes expressed in each tissue; 3) number of tissues per organism, in which a pathway is expressed; 4) pathway–tissue agreement between human and the compared organisms (JIs and PCA); 5) overview of top 100, 200 and 500 pathway–tissue combinations.

Suppl. Table 13: Results using different tissue and pathway cutoffs and experimental evidence from TISSUES. This table contains: 1) tissue confidence cutoffs and number of expressed genes; 2) average pathway expression in each tissue and organism; 3) number of pathways expressed in at least 1,3 or 7 tissues in each organism; 4) number of pathways expressed in each tissue and organism using the 50 percentile tissue confidence cutoff and different pathway cutoffs; 5) average Jaccard indices for each tissue and comparison; 6) overview of the top 200 pathway–tissue combinations.

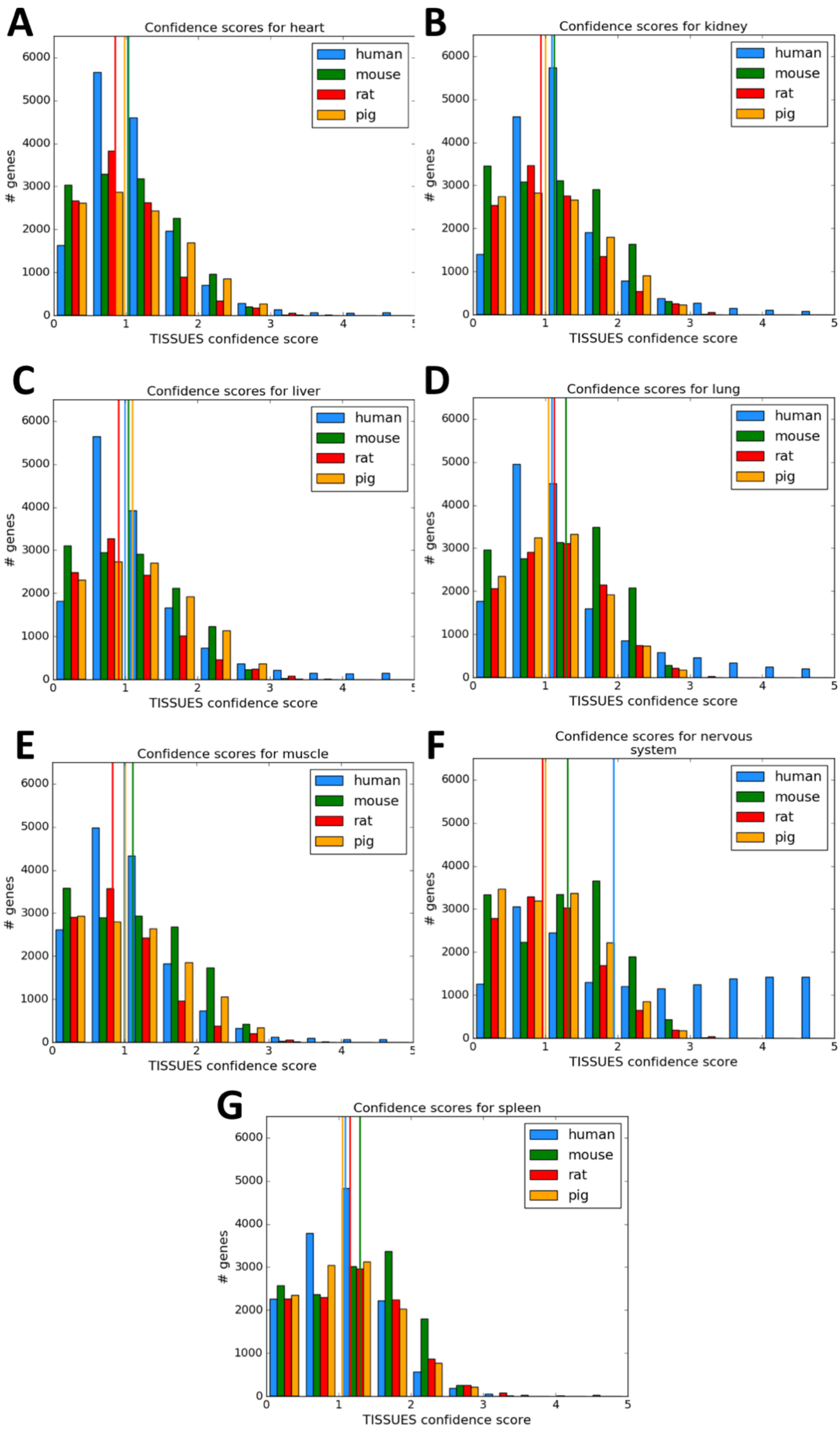

Suppl. Figure 1: Distribution of confidence scores per organism for experimental data in TISSUES: *A) heart; B) kidney; C) liver; D) lung; E) muscle; F) nervous system; G) spleen. For each tissue, the number of genes with a given score for each organism (human, mouse, rat, pig) is plotted. Straight lines indicate the 50 percentile confidence cutoffs for each organism and tissue.*

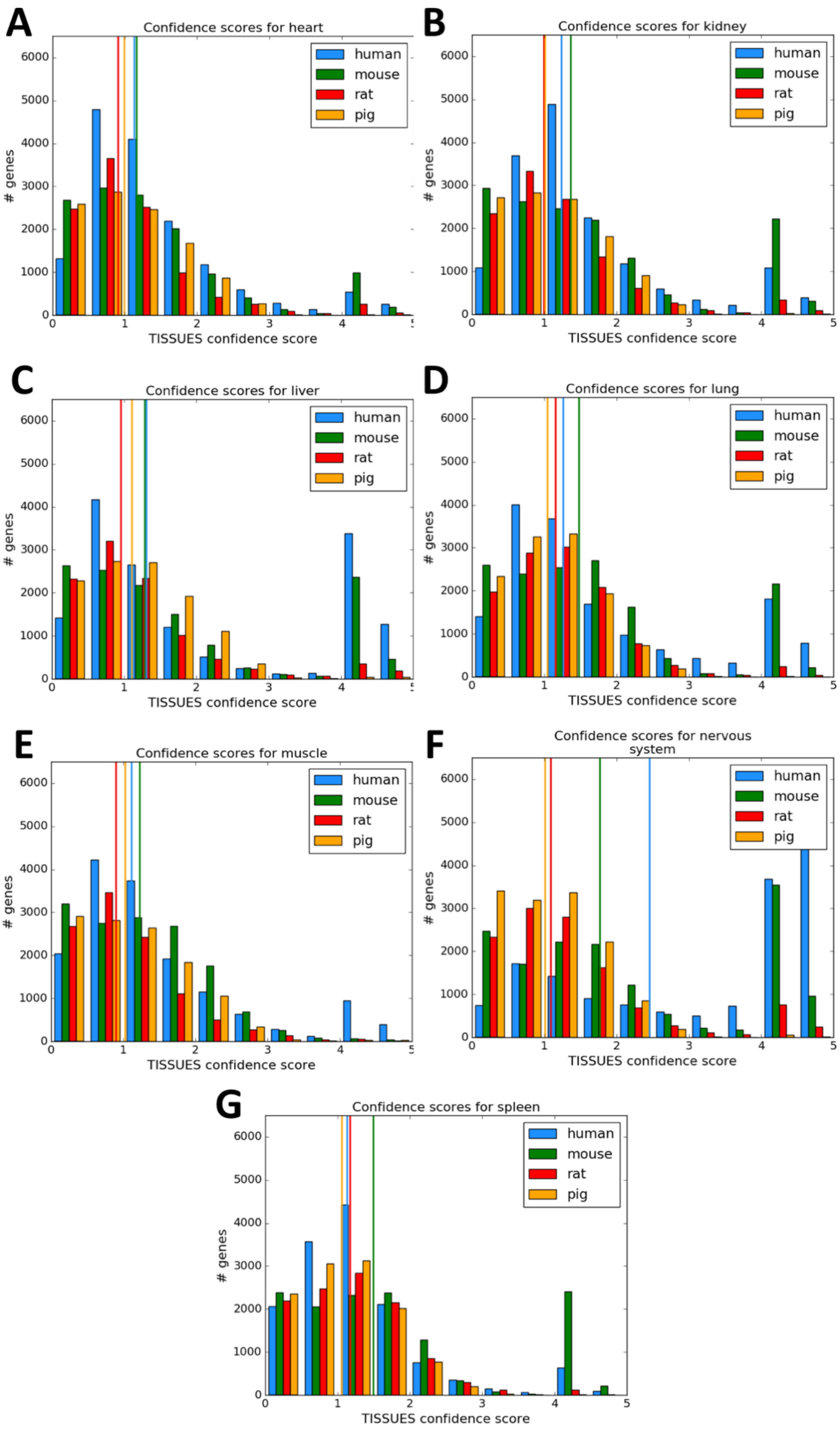

Suppl. Figure 2: Distribution of confidence scores per organism for all data in TISSUES: *A) heart; B) kidney; C) liver; D) lung; E) muscle; F) nervous system; G) spleen. For each tissue, the number of genes with a given score for each organism (human, mouse, rat, pig) is plotted. Straight lines indicate the 50 percentile confidence cutoffs for each organism and tissue.*

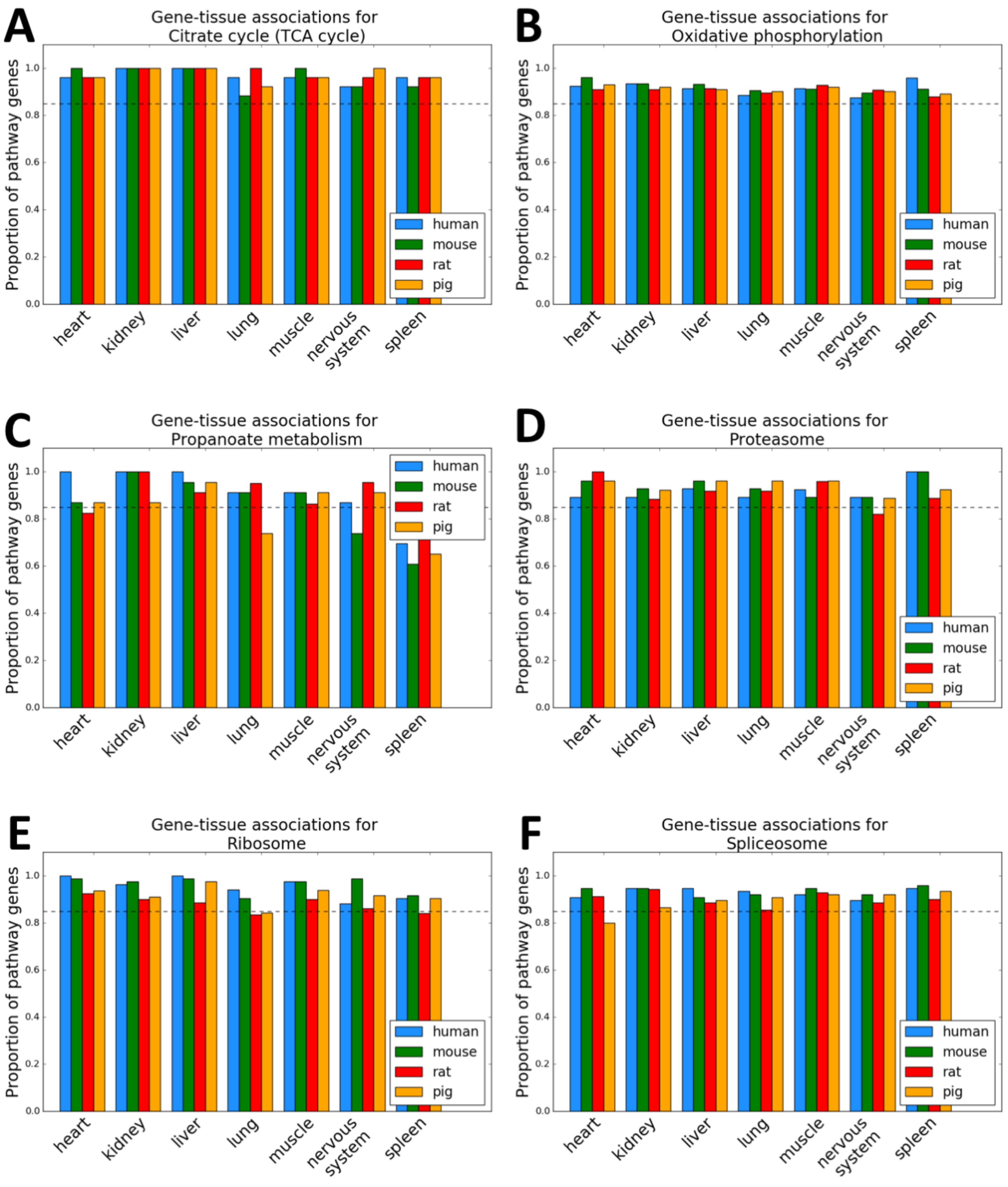

Suppl. Figure 3: Proportion of pathway genes expressed in each tissue for broadly expressed pathways. *Each panel shows the distribution for one pathway using only experimental data from TISSUES. Each bar shows the proportion of expressed pathway genes above the 50 percentile confidence cutoff compared to all pathway genes with any tissue expression information for this tissue and organism. The dotted line indicates the cutoff (0.85 or 85%) for defining a pathway as expressed.*

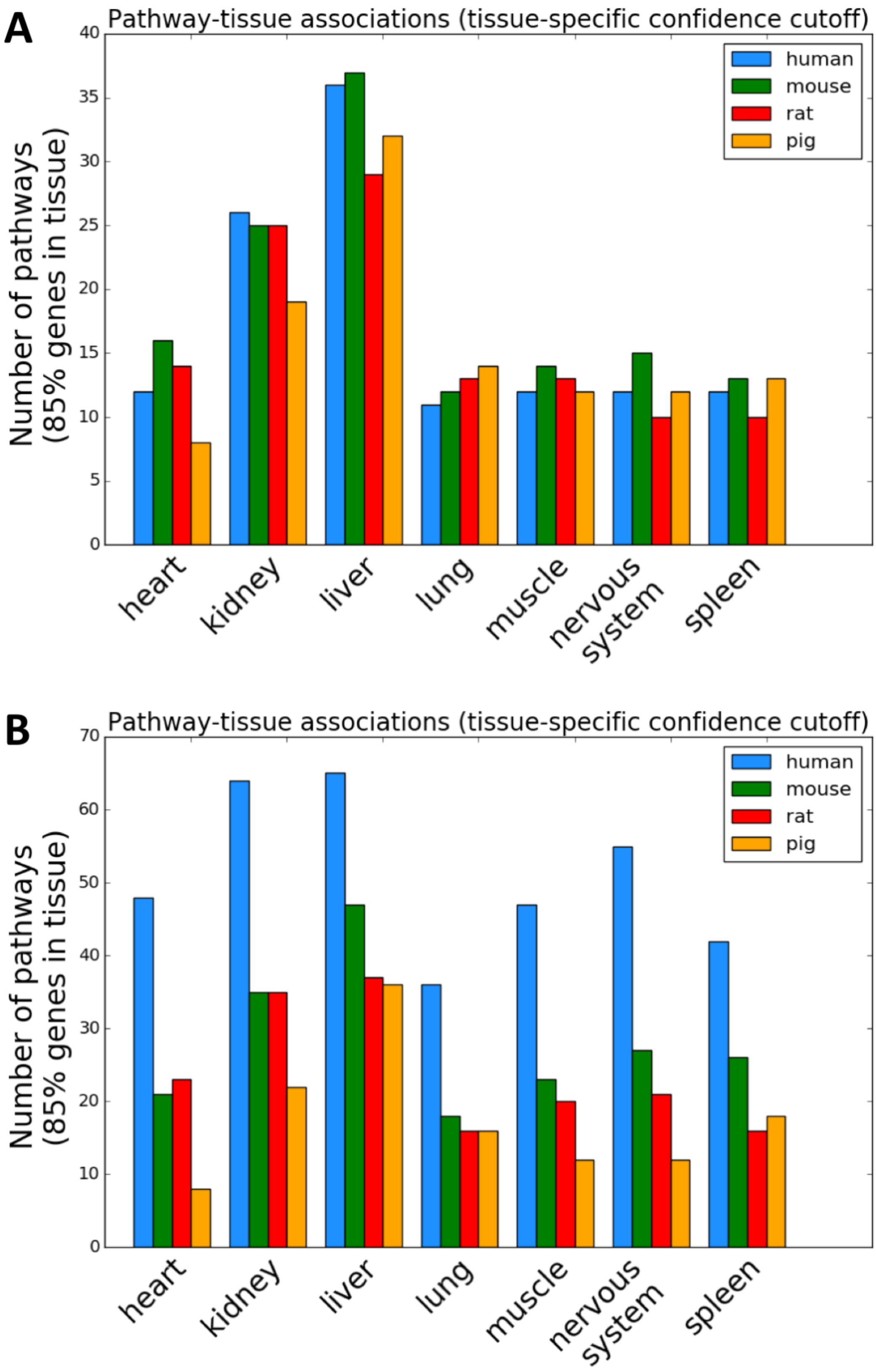

Suppl. Figure 4: Number of pathways expressed in each tissue using default cutoffs. *The plot is based on (A) the experimental data and (B) all data in TISSUES. A pathway is considered expressed in a tissue if 85% of the orthologous pathway genes with any tissue information have a TISSUES confidence score above the 50 percentile cutoff. 203 KEGG pathways and seven tissues (heart, lung, kidney, nervous system, liver, spleen, and muscle) were considered in the analysis.*

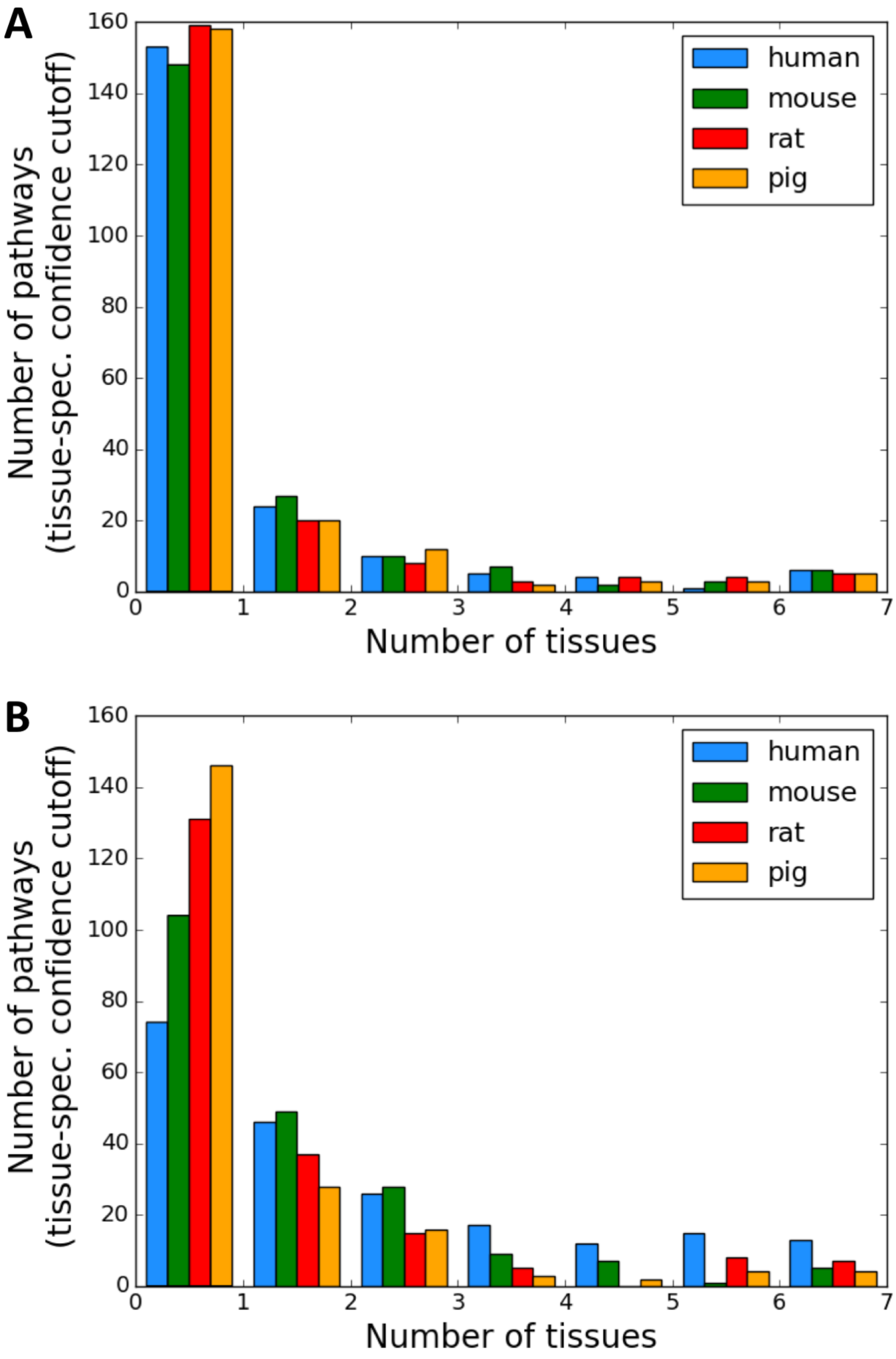

Suppl. Figure 5: Number of tissues with expressed pathways using default cutoffs. *The plot is based on (A) the experimental data and (B) all data in TISSUES. A pathway is considered expressed in a tissue if 85% of the pathway genes with any tissue information have a TISSUES confidence score above the 50 percentile cutoff. 203 KEGG pathways and seven tissues (heart, lung, kidney, nervous system, liver, spleen, and muscle) were considered in the analysis.*

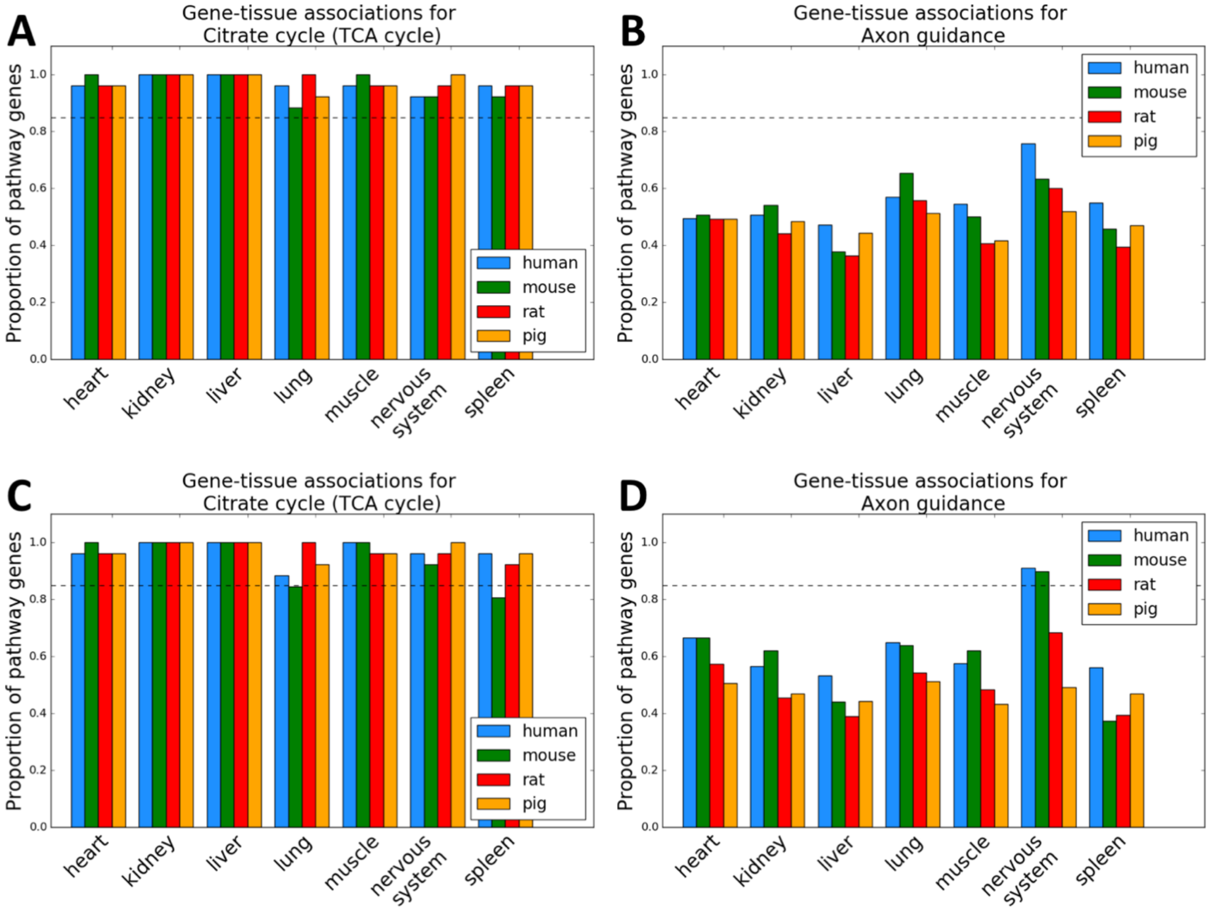

Suppl. Figure 6: Proportion of pathway genes expressed in each tissue for two selected pathways. *Citrate cycle (TCA cycle) is an example for a broadly expressed pathway, while Axon guidance is a tissue-specific pathway. Each bar shows the proportion of expressed pathway genes above the 50 percentile confidence cutoff and all pathway genes with experimental (A-B) and all (C-D) TISSUES expression information for this tissue and organism. The dotted line indicates the cutoff (0.85 or 85%) for defining a pathway as expressed.*

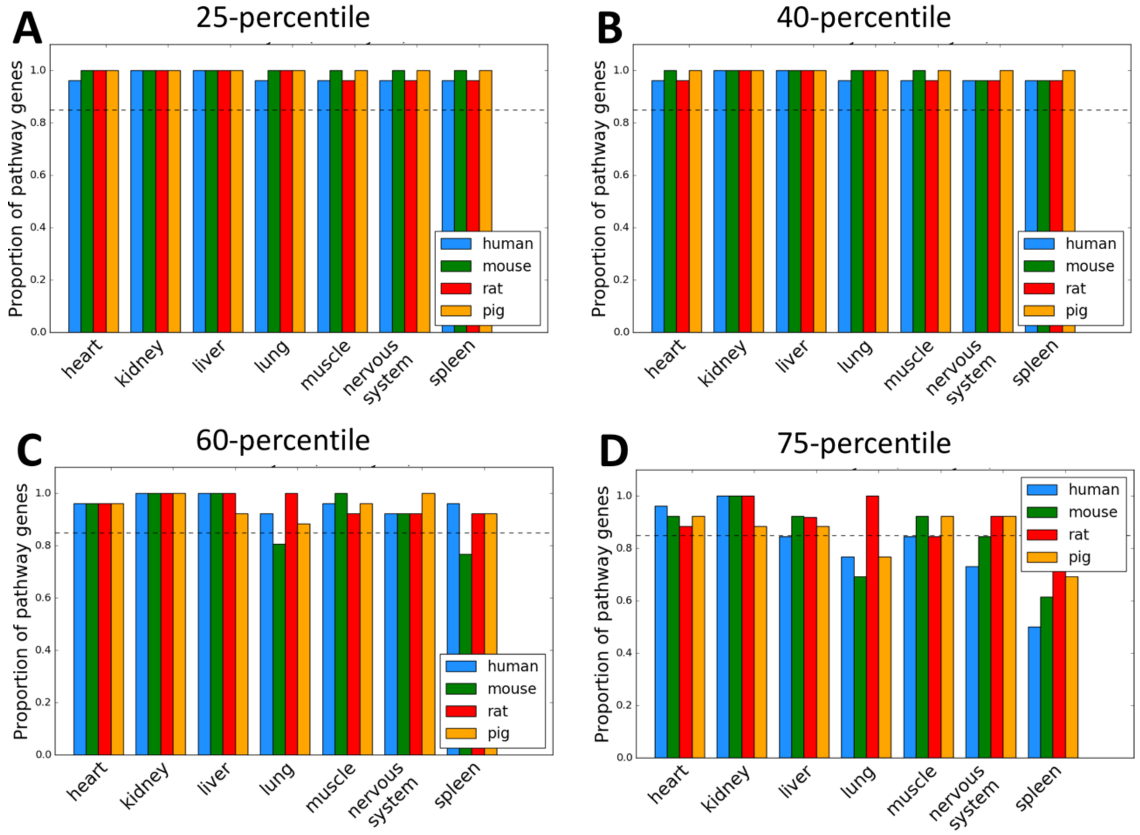

Suppl. Figure 7: Proportion of pathway genes expressed in the KEGG pathway TCA cycle using different tissue confidence cutoffs*. (A) 25 percentile; (B) 40 percentile; (C) 60 percentile; (D) 75 percentile.*

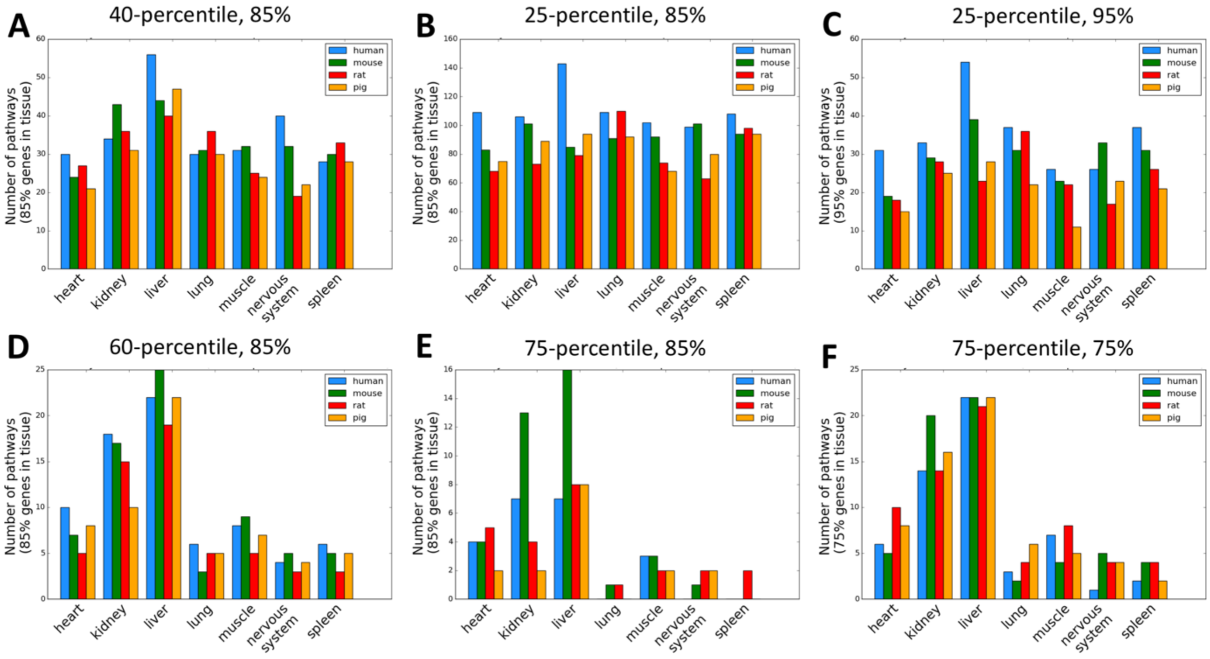

Suppl. Figure 8: Number of pathways expressed in each tissue using different cutoffs and the experimental data in TISSUES (203 KEGG pathways and seven tissues). *A pathway is defined as expressed if 85% (A-B, D-E), 95% (C), or 75% (F) of the pathway genes with any tissue information have a TISSUES confidence score above the 40 (A), 25 (B-C), 60 (D), and 75 percentile (E-F) cutoff.*

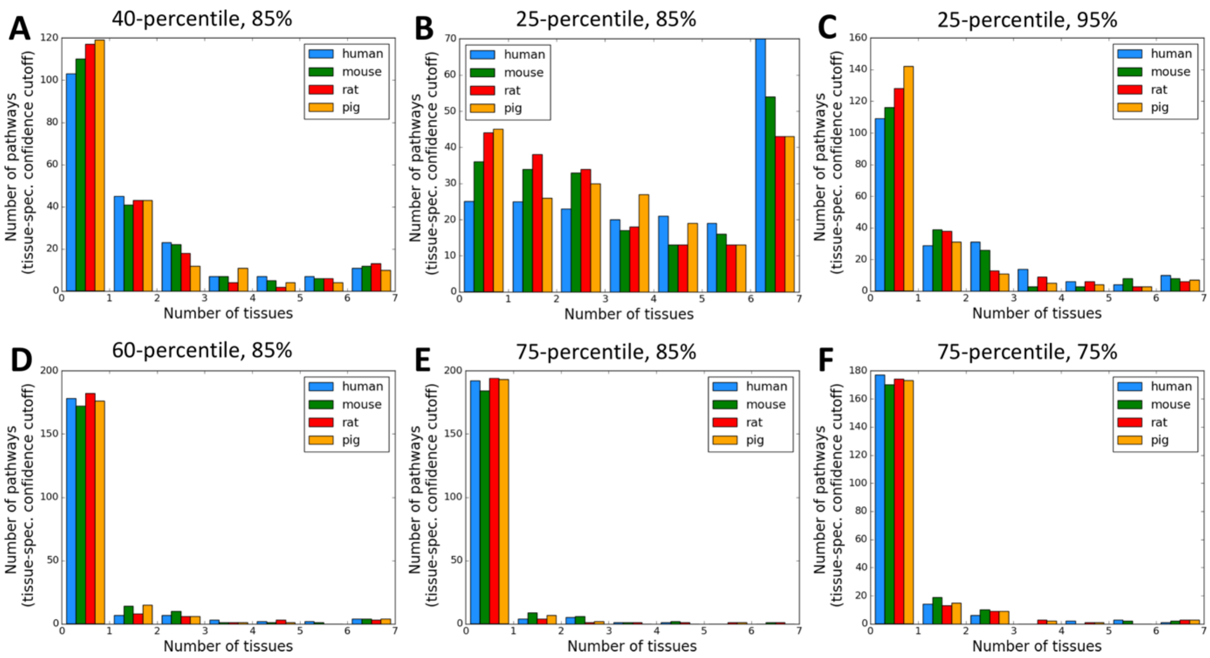

Suppl. Figure 9: Number of tissues with expressed pathways using different cutoffs and the experimental data in TISSUES (203 KEGG pathways and seven tissues). *A pathway is defined as expressed if 85% (A-B, D-E), 95% (C), or 75% (F) of the pathway genes with any tissue information have a TISSUES confidence score above the 40 (A), 25 (B-C), 60 (D), and 75 percentile (E-F) cutoff.*

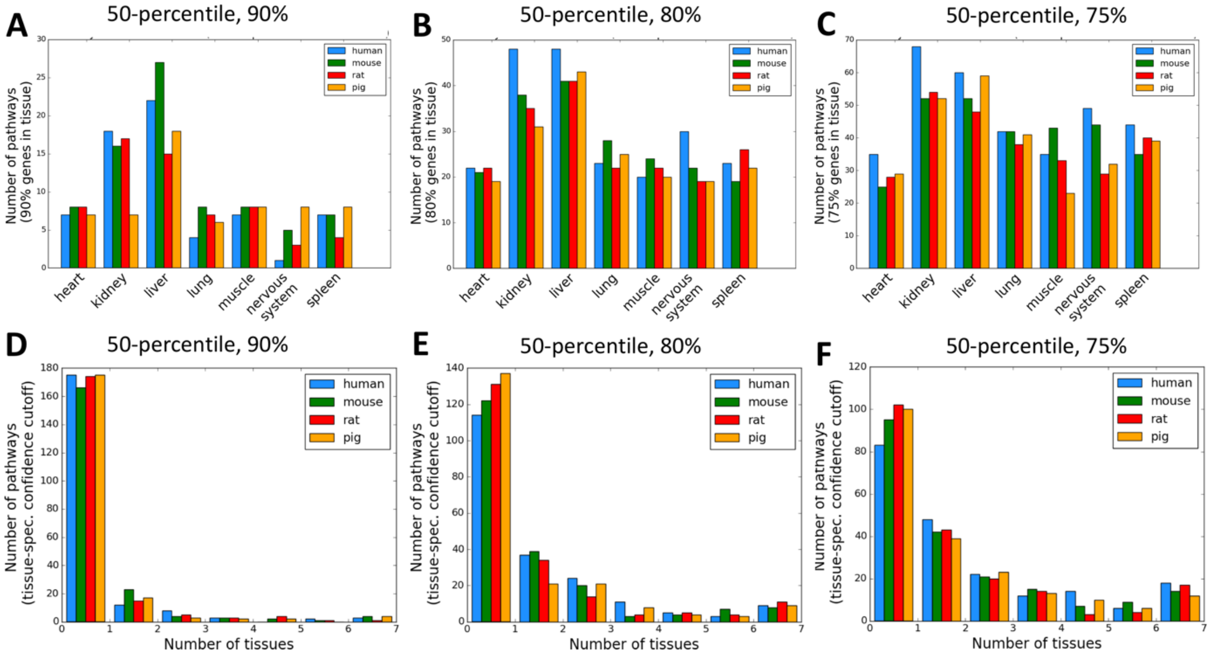

Suppl. Figure 10: Number of pathways and tissues using different pathway expression cutoffs and the experimental data in TISSUES (203 KEGG pathways and seven tissues). *A pathway is defined as expressed if 90% (A, D), 80% (B, E), or 75% (C, F) of the pathway genes with any tissue information have a TISSUES confidence score above the 50 percentile confidence cutoff.*

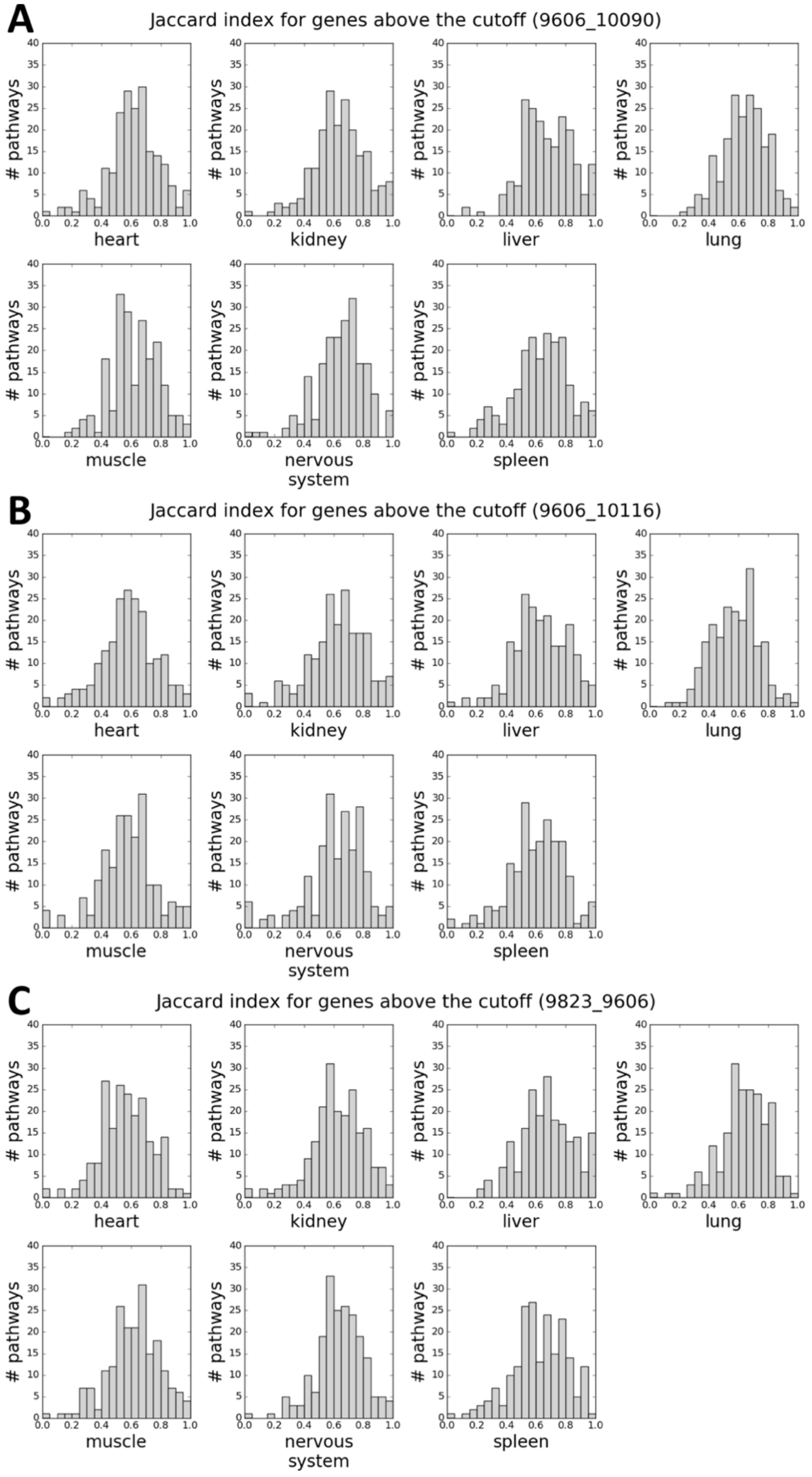

Suppl. Figure 11: Distribution of Jaccard indices for all pathways across all tissues*. The organism pairs (A) human–mouse (9606_10090), (B) human–rat (9606_10116), and (C) human–pig (9823_9606) are compared based on experimental data from the TISSUES database. Each plot represents the number of pathways (y–axis) with a given JI (x–axis) for the given tissue and organism.*

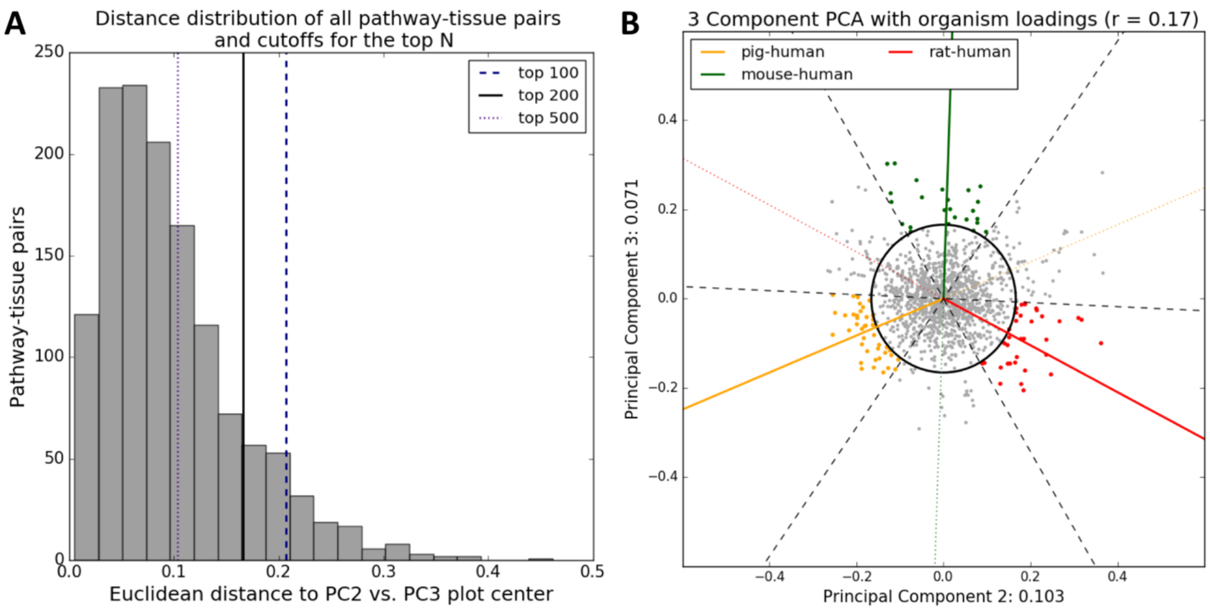

Suppl. Figure 12: Selection of pathway–tissue pairs based on PC2 & PC3. *(A) Distribution of pathway–tissue pair distances from the center of the PC2 & PC3 plot. The vertical lines indicate the cutoffs that need to be used for choosing the top 100, 200 and 500 pairs furthest away from the center. (B) The principal component (PC) analysis was performed on the Jaccard indices (JI) for all pathway–tissue pairs (grey dots) representing the comparisons mouse–human, rat–human, and pig–human using experimental data from the TISSUES database. The PCA loadings are shown as solid lines and colored by the model organism responsible for their direction. The size of the circle is chosen such that 200 pathway–tissue pairs lie outside of it. The dashed and dotted lines are used to assign pathway–tissue pairs to the respective groups: mouse, rat, pig, mouse & rat (opposite of pig), rat & pig (opposite of mouse), and mouse & pig (opposite of rat). For each loading, the pairs located close to it (between two black dashed lines) were identified and colored in the same color as the loading. The pairs locates outside of the circle and not close to any of the loadings are assigned to the groups of two organisms on their sides.*

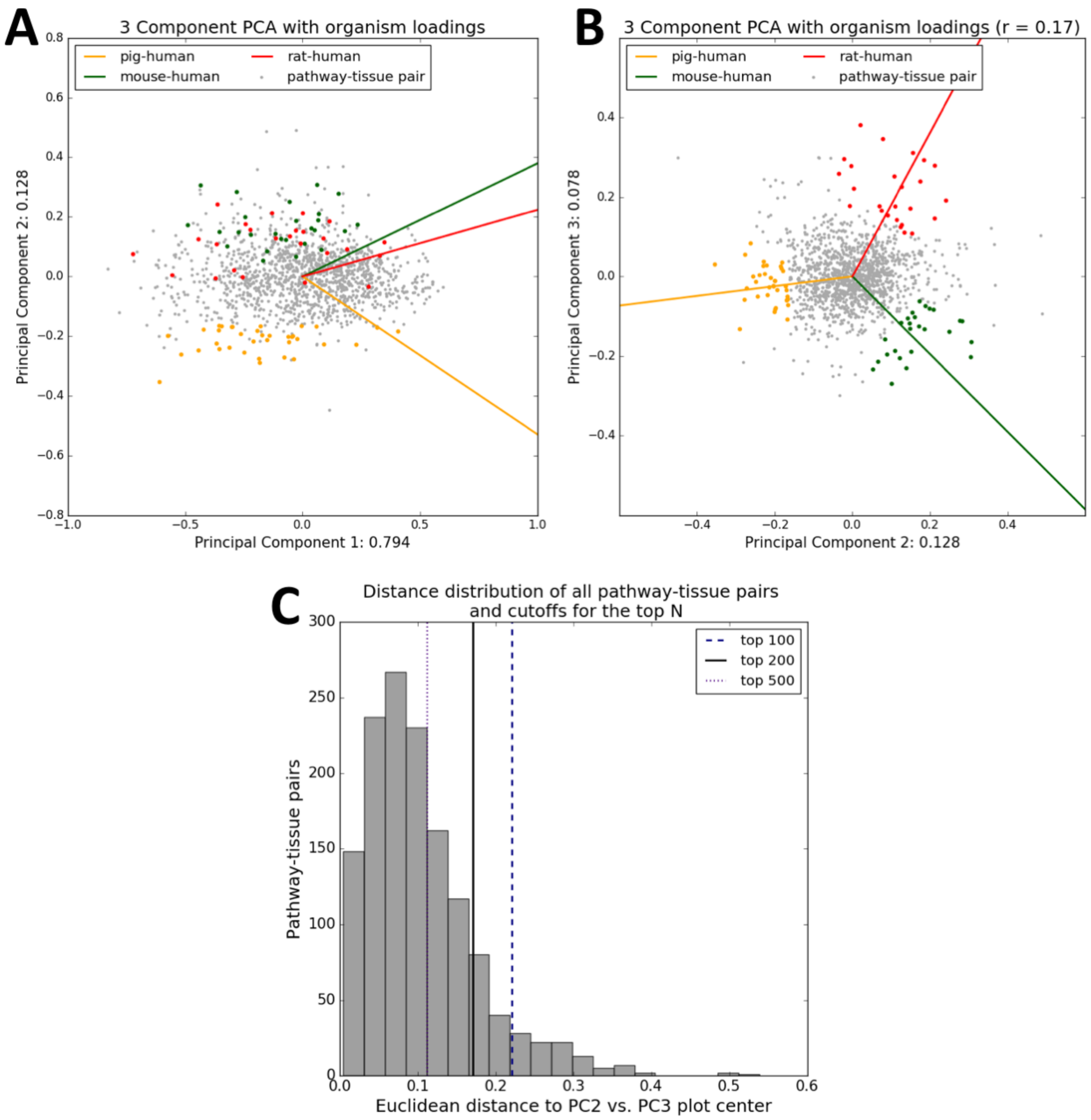

Suppl. Figure 13: Principal component analysis of the pathway–tissue agreement between human and animal models for all data in TISSUES. *(A-B) PCA was performed on the Jaccard indices (JIs) for all pathway–tissue pairs with at least 5 expressed pathway genes (grey dots) for the comparisons mouse–human, rat–human, and pig–human using all data in TISSUES. The PCA loadings are shown as lines and colored by the model organism responsible for their direction. PC1 shows a clear separation between pathway–tissue pairs with high JI, while PC2 and PC3 clearly separate the data based on the differences between the animal models (see also Suppl. Table 12). The pathway–tissue pairs located closest to each loading and furthest away from the center of the PC2 & PC3 plot are colored in the same color as the loading and assigned to the corresponding organism to indicate that for these pairs, this organism agrees more with human (see Methods section for more details). (C) Distribution of pathway–tissue pair distances from the center of the PC2 & PC3 plot. The vertical lines indicate the cutoffs that need to be used for choosing the top 100, 200 and 500 pairs furthest away from the center.*

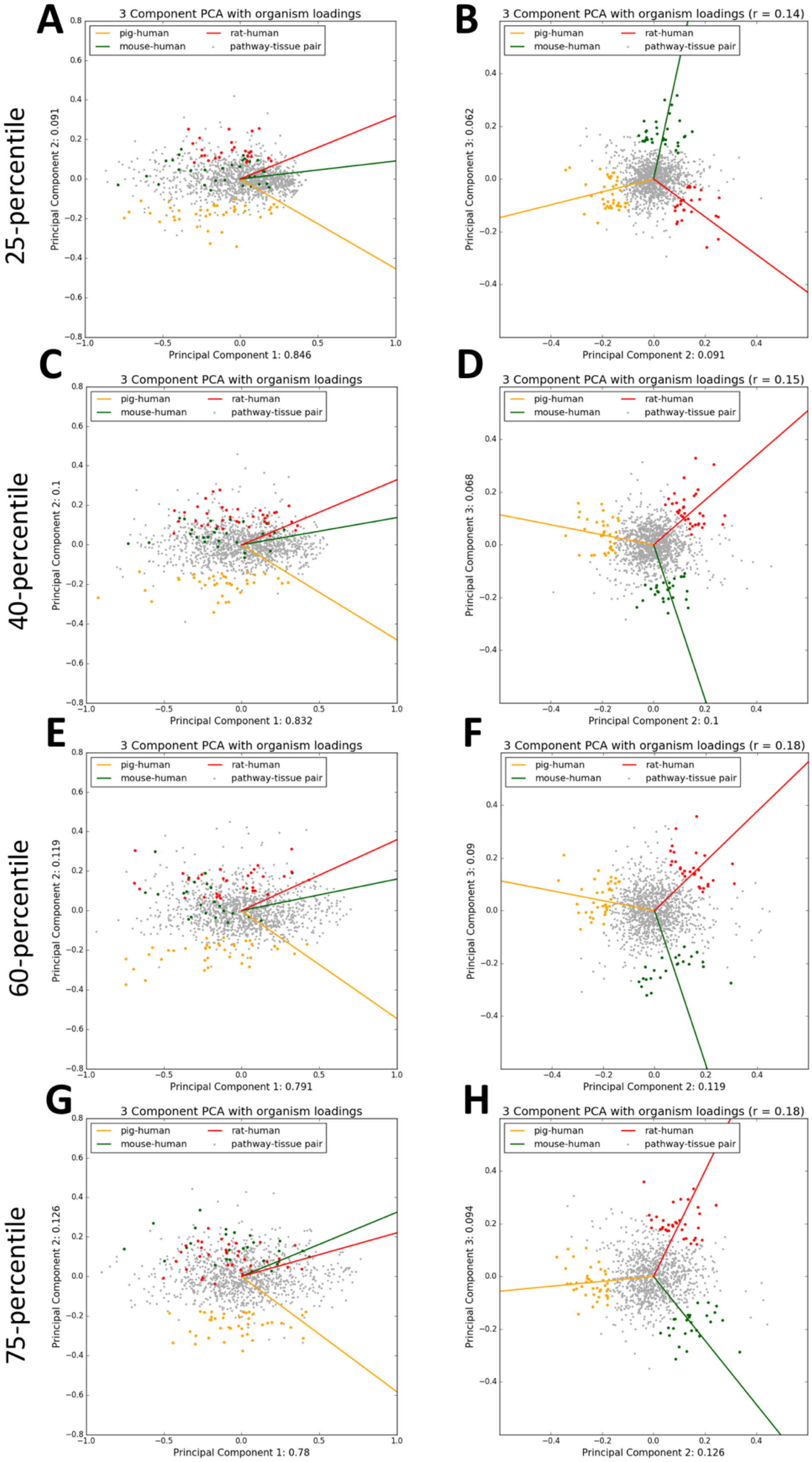

Suppl. Figure 14: Principal component analysis of the pathway–tissue agreement between human and animal models for different tissue confidence cutoffs. *(A-B) 25 percentile; (C-D) 40 percentile; (E-F) 60 percentile; (G-H) 75 percentile.*
